# Mesenchymal stem/stromal cells as a delivery platform in cell and gene therapies

**DOI:** 10.1186/s12916-015-0426-0

**Published:** 2015-08-12

**Authors:** Naomi D’souza, Filippo Rossignoli, Giulia Golinelli, Giulia Grisendi, Carlotta Spano, Olivia Candini, Satoru Osturu, Fabio Catani, Paolo Paolucci, Edwin M. Horwitz, Massimo Dominici

**Affiliations:** 1 grid.413363.00000000417695275Department of Medical and Surgical Sciences for Children & Adults, University-Hospital of Modena and Reggio Emilia, Via del Pozzo 71, 41124 Modena, Italy; 2 grid.261331.40000000122857943The Division of Hematology/Oncology/BMT, Nationwide Children’s Hospital, Departments of Pediatrics and Medicine, The Ohio State University College of Medicine, Columbus, Ohio USA

**Keywords:** Differentiation, gene therapy, microvesicles, MSC, secretion

## Abstract

Regenerative medicine relying on cell and gene therapies is one of the most promising approaches to repair tissues. Multipotent mesenchymal stem/stromal cells (MSC), a population of progenitors committing into mesoderm lineages, are progressively demonstrating therapeutic capabilities far beyond their differentiation capacities. The mechanisms by which MSC exert these actions include the release of biomolecules with anti-inflammatory, immunomodulating, anti-fibrogenic, and trophic functions. While we expect the spectra of these molecules with a therapeutic profile to progressively expand, several human pathological conditions have begun to benefit from these biomolecule-delivering properties. In addition, MSC have also been proposed to vehicle genes capable of further empowering these functions. This review deals with the therapeutic properties of MSC, focusing on their ability to secrete naturally produced or gene-induced factors that can be used in the treatment of kidney, lung, heart, liver, pancreas, nervous system, and skeletal diseases. We specifically focus on the different modalities by which MSC can exert these functions. We aim to provide an updated understanding of these paracrine mechanisms as a prerequisite to broadening the therapeutic potential and clinical impact of MSC.

**Electronic supplementary material:**

The online version of this article (doi:10.1186/s12916-015-0426-0) contains supplementary material, which is available to authorized users.

## Introduction

The therapeutic promise of multipotent mesenchymal stem/stromal cells (MSC), a population of adult stem cells that can differentiate into cells deriving from mesoderm lineage, is rising [1–3]. MSC, historically isolated from bone marrow (BM), emerged in the biomedical field for their proliferative capacity and the potential to generate skeletal-related tissues [4]. Research therefore originally focused on their ability to differentiate into committed cell types within injured areas. More recently, evidence suggests that other MSC-related mechanisms, such as secretion of cytokines or release of microvesicles (MV), may play a significant role, by promoting the stimulation of endogenous cells, the inhibition of apoptosis, neovascularization, and anti-inflammatory responses [5–7]. Early in vivo evidence suggested that MSC may also induce tolerance [8]. A large body of in vitro data subsequently supported these findings, demonstrating MSC immunosuppressive functions on different immune effectors [9]. These findings revealed that MSC retain unique immunological features, which are paving the way for their clinical application in the treatment of invalidating or deadly immune-related disorders [6, 10, 11].

These MSC secretory functions have been progressively enhanced by cell modification within gene therapy approaches, promoting tissue restoration in a more targeted manner. MSC can be modified to carry therapeutic genes, serving as programmed molecule transmitters to overcome limitations connected with direct injection of beneficial proteins. In particular, these modified MSC can be used when better bioavailability of the desired molecule is required.

The emerging role of these mechanisms in specific contexts can be considered a paradigm changer. A proper understanding of these novel actions by MSC is desirable to allow regenerative therapies to gain robust clinical importance. To date, clinical trials have shown mild or no adverse effects from MSC treatment [10, 12]. Encouraging results have led to a growing number of applications that have received tremendous attention, such as the delivery of therapeutic products to repair neural injury; the amelioration of cardiovascular events; the promotion of bone and cartilage regeneration; and for counteracting liver, pancreas, lung, and kidney disorders. This review dissects these therapeutic applications of MSC, focusing on their ability to spontaneously or artificially secrete paracrine factors to counteract still-challenging human diseases.

## The nervous system

Neurodegenerative disorders are attributed to the degeneration of specific neural cells with subsequent functional loss. Cell replacement and gene transfer to diseased or injured brain provide the basis for the development of new treatment strategies for a broad spectrum of human neurological conditions, including multiple sclerosis (MS) [13, 14], amyotrophic lateral sclerosis (ALS) [15, 16], Parkinson’s disease (PD) [17], Huntington’s disease (HD) [18], spinal cord injury (SCI) [19, 20], and stroke [21]. Growing evidence suggests that the effects orchestrated by MSC might only be marginally associated with the generation of newly graft-derived cells [22, 23], and MSC seem more likely to be producing neurotrophic and/or immunomodulatory factors to foster tissue repair in vivo (Table 1; Additional file 1: Link 1.1) [24].

**Table 1:** Links to broad spectra of MSC regenerative potential

Organ		Wild-type MSC	Gene-modified MSC
	Central and peripheral nervous systems	Additional file 1: Link 1.1	Additional file 1: Link 1.2
	Heart	Additional file 2: Link 2.1	Additional file 2: Link 2.2
	Lung	Additional file 3: Link 3.1	Additional file 3: Link 3.2
	Liver	Additional file 4: Link 4.1	Additional file 4: Link 4.2
	Pancreas	Additional file 5: Link 5.1	Additional file 5: Link 5.2
	Kidney	Additional file 6: Link 6.1	Additional file 6: Link 6.2
	Skeletal system	Additional file 7: Link 7.1	Additional file 7: Link 7.2

Adipose (AD) or BM-derived MSC reduce the disease severity in a mouse model of MS mainly by influencing the immune response. These effects are especially reported in the early stages, where the autoreactive response against myelin begins to trigger disease development [13, 14]. Similarly, MSC immunosuppression applies to other neurological models, such as Krabbe disease [25].

Early clinical trials demonstrated the safety and feasibility of MSC for MS [26, 27] and, since then, a growing number of clinical studies enforced those observations [28]. Besides the absence of major side effects following intravenous (i.v.) or intrathecal delivery, few studies also showed that MSC therapy can improve or stabilize the course of progressive MS [29]. Structural and functional improvements in visual function have also been reported in some patients [30]. Immunological changes compatible with a reduced pro-inflammatory environment have been described, and are an indirect hint of the possible MSC-mediated actions in the context of MS [31].

Aside from immunomodulation, the beneficial effect of MSC may derive from the induction of local neurogenesis through the secretion of neural growth factors, such as basic fibroblast growth factor (bFGF), platelet-derived growth factor (PDGF), and brain-derived neurotrophic factor (BDNF) [13]. These same factors have been implicated in other experimental settings, such as improving motor performance in transgenic mouse models of ALS [15, 16]. An additional mechanism was reported by Marconi et al., who hypothesized that AD-MSC derived factors influence astrocytic secretome, which in turn can amplify the impact of AD-MSC. Thus, MSC can trigger a virtuous cycle to protect neurons from deterioration in the ALS mouse model [15]. In support of this concept, within a gene therapy context (Table 1; Additional file 1: Link 1.2), Suzuki et al. forced glial cell-derived neurotrophic factor (GDNF) expression in MSC and demonstrated an improvement in motor function in vivo, along with delayed disease progression and increased life span, in a rat model of familial ALS [32].

MSC-derived neurotrophic factors can also attenuate dopaminergic dysfunction and neuronal loss in a model of neurotoxin-induced PD [17], and GDNF-overexpressing MSC placed into the striatum of a PD rat model suggested that genetic modification of MSC holds therapeutic potential for PD [33, 34]. Similarly, Dey et al. demonstrated that human BM-MSC modified to express BDNF were able to reduce neuronal degeneration in a mouse model of HD [35]. MSC were also modified by microRNA encoding sequences targeting adenosine kinase (ADK) in a background of epilepsy, providing evidence that an ADK knockdown in human MSC reduces acute injury and seizures when injected into mouse hippocampus [36, 37]. Neurotrophic factors may also contribute to the reduction of infarct volume in cerebral ischemia; vascular endothelial growth factor (VEGF), epidermal growth factor (EGF), bFGF, BDNF, and GDNF increase after MSC transplantation in rats [21]. More specifically, an in vivo study [38] showed that exogenous EGF improved cerebral ischemic condition by inhibiting free radical generation and/or lipid peroxidation, preventing neuronal damage. For this reason, EGF and other factors were forced into MSC, with favorable outcomes in stroke animal models [39–42].

Based on these pre-clinical findings, several clinical trials explored and confirmed the safety of MSC-based approaches for ischemic strokes, reporting a functional recovery and a reduction in lesion extension a few weeks after i.v. infusion [43–45]. The abundance of pre-clinical data for other neurological conditions, such as PD and SCI, prompted the employment of MSC in pilot clinical studies that generated data on the safety and feasibility of the strategy [46, 47]. Further controlled clinical studies shall now be required to validate these outcomes and generate more evidence for a solid therapeutic benefit.

Beside central nervous tissues, MSC can also promote axonal regrowth in SCI, along with the production of neutrophil-activating protein-2 and neurotrophin (NT)-3 [20]. Thus, BM-MSC have been modified for NT-3 production with partial effects in vivo [48] (Table 1; Additional file 1: Links 1.1 and 1.2).

## The heart

There is extreme clinical interest in novel treatments to improve heart function, and cellular therapies show promise [49]. Several types of cell have been so far used with the aim of generating functional cardiomyocytes and/or vascular cells in damaged myocardial tissue. MSC have progressively gained importance within these approaches [50].

**Fig. 1:**
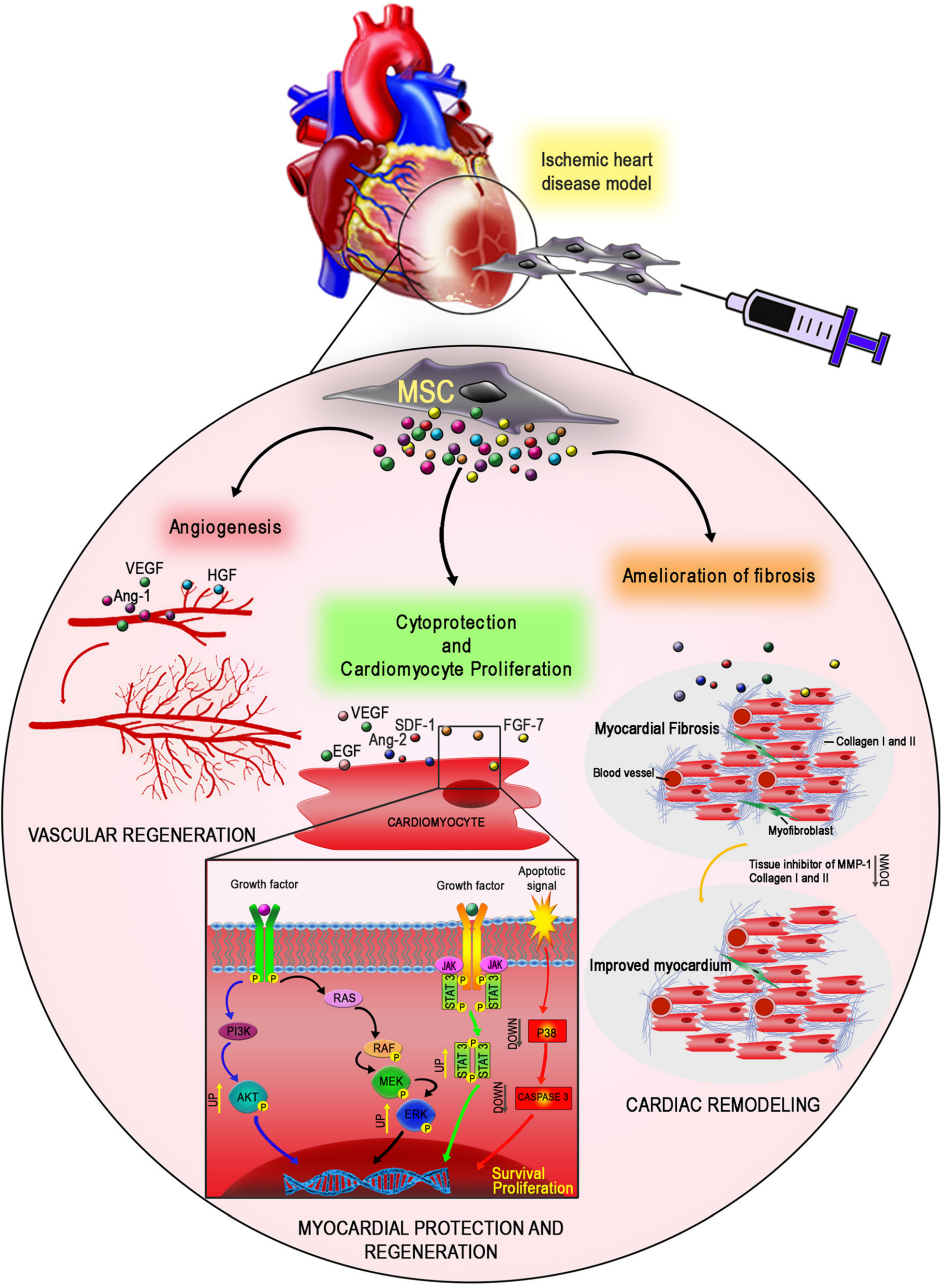
MSC paracrine action/mechanisms in heart regeneration. Soluble factors released by MSC play an essential role in the post-ischemic reparative process improving angiogenesis, cytoprotection, and endogenous cardiac regeneration and reducing fibrosis. *Ang-1* angiopoietin 1, *HGF* hepatocyte growth factor, *MSC* mesenchymal stem/stromal cells, *VEGF* vascular endothelial growth factor

Differentiation of transplanted MSC into cardiomyocytes and vessels was originally proposed as the main mechanism underlying their therapeutic action in cardiovascular diseases [51, 52]. More recently, it has been shown that the number of newly generated cells is too low to justify functional improvements, and evidence supports the hypothesis that paracrine mechanisms mediated by MSC may play an essential role in the reparation (Table 1; Additional file 2: Link 2.1) [53]. The mechanisms mediating these effects by paracrine factors are numerous and not completely clear, although it has been demonstrated that they can lead to neovascularization, cytoprotection, and endogenous cardiac regeneration (Fig. 1). More, post-infarction inflammatory and fibrogenic processes, cardiac contractility, and cardiac metabolism may also be influenced in a paracrine fashion [49].

The complexity of MSC secretome is hindering a definitive understanding; however, clues on the biological drivers for cardiac regeneration have been emerging and consistent evidence begins to indicate some pivotal players. VEGF is emerging as a critical paracrine factor for MSC-mediated cardioprotection. Several MSC types may also differentially release insulin-like growth factor (IGF)-1, transforming growth factor (TGF)-β2, and EGF [54–56]. AD-MSC are able to secrete numerous angiogenic, arteriogenic, chemotactic, and anti-apoptotic growth factors; for this reason their secretome has been involved in a series of novel strategies to enhance tissue restoration by increased angiogenesis [57–59]. Schenke-Layland et al. showed that AD-MSC accelerated vascularization in infarcted areas, increasing both capillary and arteriole density as a result of paracrine signaling [60]. This mechanism has been supported by other investigators who have considered adult stem cells from other sources administered into animal models post myocardial infarction (MI) [58, 61, 62].

Other cytoprotective factors such as hepatocyte growth factor (HGF) and angiopoietin (Ang)-1 are released by MSC when delivered into an acute MI rat model, and are associated with a significant improvement in cardiac function through increased angiogenesis and decreased infarct size [60, 63, 64]. Similarly, Li et al. showed an increase in capillary density along with significantly higher VEGF mRNA and protein levels after AD-MSC treatment [55].

Starting from these early understandings of MSC paracrine effects within infarcted regions, several authors selected putative beneficial factors to be introduced in a gene therapy approach (Table 1; Additional file 2: Link 2.2). A promising strategy to treat MI comes from Gao et al., who overexpressed VEGF in rat BM-MSC and generated effective myogenesis, preventing progressive heart dysfunction [65]. Similarly, murine BM-MSC modified by VEGF and/or HGF improved ventricular ejection function and reduced scar size [66]. Others showed that Ang-1 genetically modified rat BM-MSC were able to improve heart function by decreasing infarct area and promoting heart remodeling [67], indicating MSC-based gene therapies as feasible tools for heart regeneration.

Besides pro-angiogenic effects, data have demonstrated that cellular benefits might also be mediated by the activation of survival kinase pathways in response to MSC-secreted cytokines, additionally suggesting prevention of programmed cell death. Such pathways include activation of Akt, extracellular signal-regulated kinase 1/2 (ERK1/2), and signal transducer and activator of transcription 3, and inhibition of p38 mitogen-activated protein kinase, all instrumental in the promotion of cell proliferation [54]. To support this concept after MI, Gnecchi et al. genetically modified rat BM-MSC with Akt, showing that metabolism, glucose uptake, and cytosolic pH were maintained, and cardiac metabolism remodeling was prevented [68].

Emerging aspects from these pre-clinical findings are also related to cell homing and tissue persistence. Both aspects are crucial for clinical outcome in both intra-vessel and intra-MI injections. Considering rat BM-MSC, researchers have demonstrated that by overexpression of C-X-C chemokine receptor type 4 (CXCR4) i.e the stromal cell-derived factor (SDF)-1 receptor (largely involved in progenitor homing and survival) was possible to enhance engraftment within the infarct, thereby improving function and promoting neo-myoangiogenesis [69]. On tissue retainment and survival, recent data revealed that overexpression of cytoprotective proteins capable of enhancing expression of pro-survival genes, such as heme oxygenase-1, is associated with an increase in MSC survival [70].

Inflammation is a detrimental factor for tissue regeneration after MI. Attention has therefore been paid to anti-inflammatory approaches based on MSC. A paradigmatic example comes from the studies of Lee et al., who investigated gene expression of MSC trapped in lungs after i.v. injection in a mouse model of MI [71]. From all identified factors, tumor necrosis factor (TNF)-stimulated gene-6, a known anti-inflammatory molecule, contributed significantly to the amelioration of heart function, reducing infarct size and improving heart remodeling.

A large number of clinical trials have been completed for cardiovascular regeneration and their outcomes published (for extensive revisions see [10, 72]), with results suggesting at least the safety of these approaches. However, emerging data from patients with chronic/acute MI and refractory angina are still contradictory, showing either no significant effects or improvements in cardiac function associated with a reduction of scar tissue [73–76]. Therefore, basic investigations are currently following these studies to provide a better understanding of the optimal MSC source, delivery manner, cell doses, cell persistence, and precise mechanisms of action to ultimately create a more favorable prospect for the clinical uses of MSC for cardiovascular disorders.

## The lung

While less prevalent than cardiovascular diseases, several lung pathologies represent unmet clinical needs possibly requiring novel cell based-therapeutic interventions. Investigations into different pulmonary diseases (Table 1; Additional file 3: Link 3.1) have revealed a basic understanding of the possible approaches for treatment that is yet to be consolidated by further pre-clinical research.

Studies on pulmonary fibrosis demonstrate that i.v. and endotracheal administration of MSC attenuates lung injury and fibrosis, suggesting a potential clinical application of MSC for the treatment of lethal idiopathic pulmonary fibrosis [77–79]. The mechanisms of the MSC-mediated amelioration in pulmonary fibrosis are not completely clear and an active participation of MSC through differentiation into alveolar epithelial cells in lung regeneration is still under debate [77, 78]. An endotoxin-induced lung fibrosis model in mice showed an MSC-mediated reduction in pulmonary fibrosis via paracrine downregulation of pro-inflammatory responses by reducing TNF-α and macrophage inflammatory protein (MIP)-2 while increasing the anti-inflammatory interleukin (IL)-10 [80]. Additionally, MSC can also upregulate matrix metalloproteinases (MMP), favoring the establishment of a microenvironment prone to extracellular matrix degradation and fibrosis reduction [77].

In a different context, a syngeneic model of pulmonary emphysema demonstrated that rat MSC reduced apoptosis of alveolar epithelial cells through upregulation of anti-apoptotic B-cell lymphoma (Bcl)-2 gene [81]. Moreover, Akram and colleagues showed that human MSC displayed site-specific migration into alveolar wounds where they secreted paracrine components for alveolar and small airway epithelial wound repair [82]. These paracrine effectors include fibronectin and lumican, known to be involved in corneal, skin, and mucosal healing. In relation to these findings, researchers have additionally gene-modified MSC for lung disorders (Table 1; Additional file 3: Link 3.2), selectively overexpressing either Ang-1 or IL-10. This resulted in a reduction of pro-inflammatory cytokines, increased lung permeability, and improved lung injury in vivo [83–85]. Experimental models of bronchopulmonary dysplasia (BPD) have also been considered; MSC mitigated lung inflammation, preventing lung vascular damage and alveolar growth impairment, ultimately inhibiting lung fibrosis [86].

Very curiously, the use of conditioned media from MSC has been shown to protect alveolar epithelial and lung microvasculature endothelial cells from oxidative stress, prevent oxygen-induced alveolar growth impairment, and stimulate endogenous lung progenitors such as bronchoalveolar stem cells [87]. For this reason, several researchers investigated the role of an emerging class of cell-derived particles, such as MV. Recent evidence suggested that MV can reduce lung inflammation and protein permeability, which in turn prevented the formation of pulmonary edema in *Escherichia coli* endotoxin-induced acute lung injury [88]. In a mouse model of hypoxic pulmonary hypertension, MV derived from mouse MSC-conditioned medium prevented vascular remodeling and an elevation in right ventricular systolic pressure by suppressing the hypoxic pulmonary influx of macrophages and by inducing pro-inflammatory and pro-proliferative mediators [89]. These data are generating a novel paradigm of tissue restoration by cell-derived bio-products that shall require far deeper investigation to determine the active principle(s) associated with the biological observations in pre-clinical models and in humans. To achieve this goal, several clinical pilot studies are already ongoing for the treatment of BPD, pulmonary emphysema, and pulmonary fibrosis (data extrapolated from www.ClinicalTrials.gov).

## The liver

The liver has remarkable regenerative capacity in response to acute injuries. Either hepatic progenitors, the oval cells, or mature hepatocytes are able to re-enter the cell cycle to restore the hepatic mass. However, under chronic damage, these cells lose their ability to regenerate, causing “liver failure” [90]. For this reason, BM-MSC and human umbilical cord (hUC)-derived MSC-based approaches were introduced in early clinical studies [91, 92] for cirrhosis and end-stage liver failure, with improvements in liver function, reduced ascites, and no safety concerns [93–95].

Several studies on animal models reported the beneficial effect of MSC in promoting hepatic regeneration or preventing pathological changes (Table 1; Additional file 4: Link 4.1). The following mechanisms have been proposed to explain this therapeutic effect: homing and differentiation into hepatocytes, secretion of trophic molecules, and suppression of inflammation [96]. The liver homing properties of MSC were confirmed by the demonstration that CXCR4 overexpression enhanced engraftment and improved early liver regeneration [97]. However, reports supporting differentiation of MSC into hepatocytes are controversial [98, 99], so authors began to explore the therapeutic potential of MSC, hypothesizing their ability to produce bioactive factors [100]. These factors include HGF, VEGF, and nerve growth factor, which have the intrinsic ability to support hepatocyte proliferation and thereby facilitate the breakdown of fibrosis [96, 100–104]. To test this hypothesis, Ishikawa et al. genetically increased the expression of HGF in MSC. They observed that modified MSC accumulated in the liver, resulting in a decrease in fibrosis in vivo, thus confirming the homing potential and therapeutic benefits of MSC towards liver fibrosis [105]. Similarly, others introduced gene therapy approaches as outlined in Additional file 4: Link 4.2. MMP and fibrinogen-like protein-1 are also reported to be upregulated, further indicating the potential of MSC to counteract cirrhosis [106, 107]. Interestingly, MSC are also likely to exert an antioxidative action on resident cells by increasing superoxide dismutase activity and inhibiting reactive oxygen species production [108].

The anti-fibrogenic action of MSC has also been enhanced by gene modification. Li et al. overexpressed human alpha-1 antitrypsin, demonstrating that gene-modified MSC engraft into recipient livers and contribute to liver regeneration without eliciting an immune response in vivo [109]. This lack of significant immune response recalls known immunomodulatory properties of MSC that may represent a significant step in restoring liver injury. The local downregulation of pro-inflammatory cytokines and upregulation of anti-inflammatory cytokines, such as IL-10, after MSC transplantation has been reported to significantly improve function and reduce fibrosis [96, 100, 103, 107, 110].

As for lungs, MSC-derived MV are gaining interest in pre-clinical models of liver injury. MSC-MV reversed CCl_4_-induced injury in mice, through the activation of proliferative and regenerative responses. These in vivo beneficial effects confirmed the in vitro findings where MV sustained higher hepatocyte viability after injuries caused by acetaminophen and H_2_O_2_. The higher survival rate in vivo in the MV-treated group was also associated with upregulation of the priming-phase genes (coding for IL-6, TNF-α, and MIP-2) during liver regeneration, which subsequently lead to higher expression of proliferation proteins, such as proliferating cell nuclear antigen and cyclin D1 [111]. In another recent study, MV derived from hUC-MSC were used to treat CCl_4_-induced mouse liver fibrosis, ameliorating liver injury by inactivating the TGF-β1/Smad signaling pathway and inhibiting the epithelial–mesenchymal transition of hepatocytes [112].

## The pancreas

The replacement of functional pancreatic β cells together with immunomodulation is seen as an attractive potential therapy for type 1 diabetes (T1D) [113]. However, extensive application of islet transplantation is hampered by the scarcity of donor tissue, the need for toxic lifelong immunosuppressive drugs, and graft failure usually within a few years [114, 115]. A possible solution to the cited challenges of islet transplantation has been found in MSC, and early trials based on their administration in patients with T1D have been reported. Carlsson et al. showed that autologous MSC treatment of new onset T1D may be a safe and feasible strategy to intervene in the disease process to preserve β-cell function. A randomized and double-blind phase II study is ongoing to validate these encouraging results [116].

From early studies, MSC immunomodulatory and paracrine properties, as opposed to the regenerative properties, are considered to have the greater effect in preventing, arresting, or reversing autoimmunity and ameliorating innate/alloimmune graft rejection (Table 1; Additional file 5: Link 5.1) [113]. At pre-clinical level, independent studies have proven that systemic MSC administration results in a functional recovery and normoglycemia [117–120]. However, the relative mechanisms contributed by MSC to this therapeutic effect are poorly understood. Reported data showed that MSC could differentiate into insulin-producing cells in vitro, indicating that transplantation of these islet-like cells is able to ameliorate hyperglycemia in diabetic rats [113, 121]. However, MSC differentiation has yielded contradictory results, mostly due to the discrepancy between the low level of functional integration of donor MSC and the observed recovery of pancreatic islets [118]. Therefore, the outcome of MSC treatment in diabetes is likely to be achieved by paracrine mechanisms along with immunomodulatory properties that can stimulate β cell repair/regeneration and abrogate immune injury, rather than by direct differentiation into β cells [113].

Although the potential trophic effect of MSC on pancreatic islets is not entirely clear, several MSC-secreted factors, such as IL-6, VEGF-A, HGF, and TGF-β, seem to improve islet cell viability and function by inhibiting apoptosis, inducing β cell proliferation, enhancing β cell insulin response to high glucose, and promoting islet revascularization [122]. Gao et al. demonstrated that conditioned medium from MSC exerts a striking protective effect on isolated islets exposed to streptozotocin (STZ). Moreover, injection of MSC-conditioned media into diabetic mice is able to partly restore the numbers of islets and β cells, and this stem cell paracrine action has been linked to activation of Akt signaling [123].

**Fig. 2:**
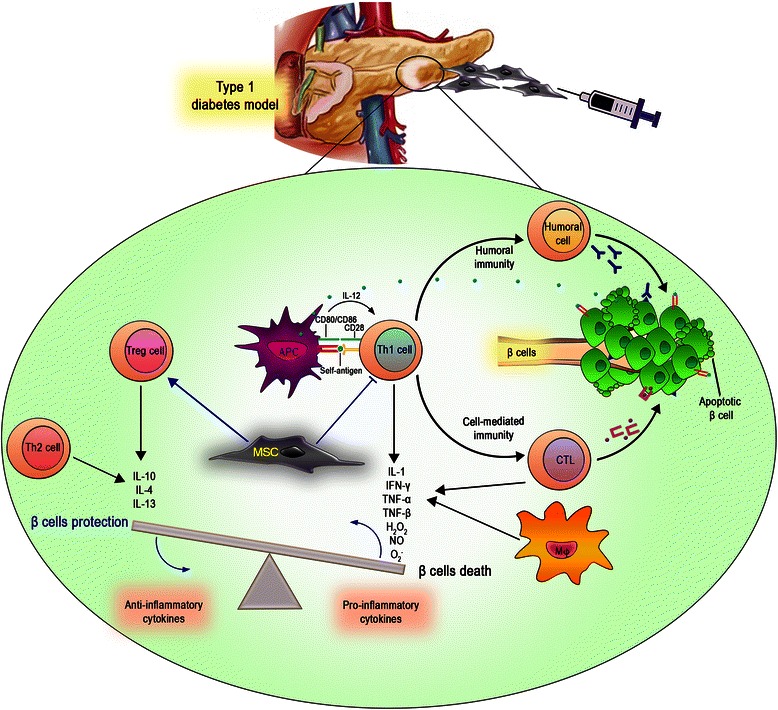
MSC immunomodulatory properties in pancreatic regeneration. MSC are able to modulate the autoimmune response in T1D either by inducing regulatory T cells or by shifting the cytokine profile from a pro-inflammatory to an anti-inflammatory one. *APC* Antigen Presenting Cell, *CTL* Cytotoxic T Lymphocyte, *Th1/2* T helper cell type 1/2, *Treg* regulatory T cell

Besides these trophic functions, MSC immunomodulatory potential has recently gained interest for the treatment of T1D, mainly as an alternative to immunosuppressive drugs (Fig. 2). Ezquer et al. observed that, after i.v. administration in STZ-induced diabetic mice, MSC engraft into secondary lymphoid organs, inhibiting self-reacting T-cell expansion either by inducing regulatory T cells (Tregs) or by shifting the cytokine profile from a pro-inflammatory to an anti-inflammatory one [118]. Similarly, following MSC i.v. injection into NOD mice, others observed an increase in the percentage of Tregs and a shift towards a Th2 cytokine profile, both of which have been shown to help the recovery of islet cells [124].

The recent work by Favaro et al. provides in vitro evidence that some of the immunomodulatory actions of BM-MSC can be vicariated by MSC-derived MV. The observed MV inhibitory effect on glutamic acid decarboxylase (GAD)-stimulated peripheral blood mononuclear cells (PBMC) seems to involve prostaglandin E2 and TGF-β signaling pathways and IL-10. Blockade of MV internalization into PBMC, as well as pre-treatment of MV with RNAse, reduced IL-10 and TGF-β1 transcripts in MV-treated PBMC stimulated with GAD65. Furthermore, levels of mir-21, known to enhance TGF-β signaling, were increased in GAD-stimulated PBMC in the presence of MV [125]. To evaluate this hypothesis, a clinical trial with i.v. infusion of hUC-MSC-MV is ongoing to reduce inflammation and improve the β cell mass in patients with T1D (www.ClinicalTrials.gov: NCT02138331).

MSC have also been genetically modified to reprogram them into a pancreatic β cell lineage as well as to serve as gene delivery vehicles (Table 1; Additional file 5: Link 5.2) [126, 127]. For instance, gene-modified MSC carrying the human insulin gene have been assessed for T1D therapy [128]. Moreover, gene-modified BM-MSC carrying IL-1 receptor antagonist together with HGF or VEGF demonstrated clear advantages in improving the outcome of islet transplantation compared to non-transduced BM-MSC [129, 130]. Besides the potential for MSC to ameliorate T1D outcome, MSC have been evaluated for metabolic control in experimental models of type 2 diabetes (T2D). Si et al. proved that MSC infusion could partially reconstruct islet function and effectively ameliorate hyperglycemia in T2D rats, additionally acting to improve insulin sensitivity by upregulating glucose transporter type 4 expression and elevating phosphorylated insulin receptor substrate 1 and Akt levels in target tissues [131]. A preliminary clinical study involving 22 patients with T2D showed that Wharton’s jelly-derived MSC are able to significantly improve β-cell function without adverse effects [132].

MSC have also been evaluated as a cell-based therapeutic strategy for the treatment of other pancreatic diseases, such as acute pancreatitis (AP). Jung et al. significantly reduced the production of pro-inflammatory cytokines, increasing the production of anti-inflammatory factors by MSC. Curiously, Tregs were specifically recruited into the pancreas and along with MSC induced immunomodulation during AP [133].

## The kidney

Several studies have reported multiple beneficial effects of MSC infusion in acute kidney injury (AKI) [134]. Systemically delivered MSC home to kidney after renal injury under the control of several factors, such as SDF-1 and PDGF [135, 136]. In addition, hyaluronic acid was shown to recruit exogenous MSC to injured renal tissue, and enhanced renal regeneration via CD44 [137]. Others report that MSC infusion is followed by an accelerated recovery of renal function compared to non-treated mice. Infused cells were able to partially differentiate into endothelial or smooth muscle cells and contributed to angiogenesis, vasculogenesis, and endothelial repair (Table 1; Additional file 6: Link 6.1) [138]. However, recent data showed that this apparent reparative function of MSC could also be achieved via intraperitoneal injections of MSC-conditioned medium alone, suggesting that MSC may additionally provide paracrine factors with positive impacts on kidney injury [139]. In contrast to these findings, others observed that i.v. infusions of MSC, but not of their conditioned medium, were associated with both the rapid recovery of kidney function and the enhanced survival of the mice [140].

Although extensive clinical studies are still limited in this context, the very interesting results obtained in pre-clinical steps prompted the translation of MSC-based treatments into humans. Preliminary results in a phase I trial using supra-renal aortic injection of allogeneic BM-MSC showed the safety of allogeneic MSC delivery and an amelioration of AKI [141]. Another phase II trial (NCT 01602328) is ongoing to assess the safety and efficacy of MSC in patients developing AKI. An overall view of currently available early data confirms the safety of these treatments. Additional investigations are now required to identify the precise mechanisms of action and confirm a benefit in human kidney disorders [142].

While the exact nature of the putative beneficial factors for the kidney is still under investigation, early evidence points toward IGF-1, HGF, EGF, VEGF [139, 143, 144] and bone morphogenetic protein (BMP)-7 as players capable of restoring kidney function and protecting against fibrosis [145]. To examine these findings, IGF-1 and erythropoietin (EPO) production were enhanced in MSC by gene modification. MSC co-expressing EPO and IGF-1 improved hematocrit levels and heart function in a renal failure mouse model [146]. Similarly, Zhen-Qiang et al. demonstrated that MSC overexpressing BMP-7 were able to improve renal function and regenerate tubular cells [147].

**Fig. 3:**
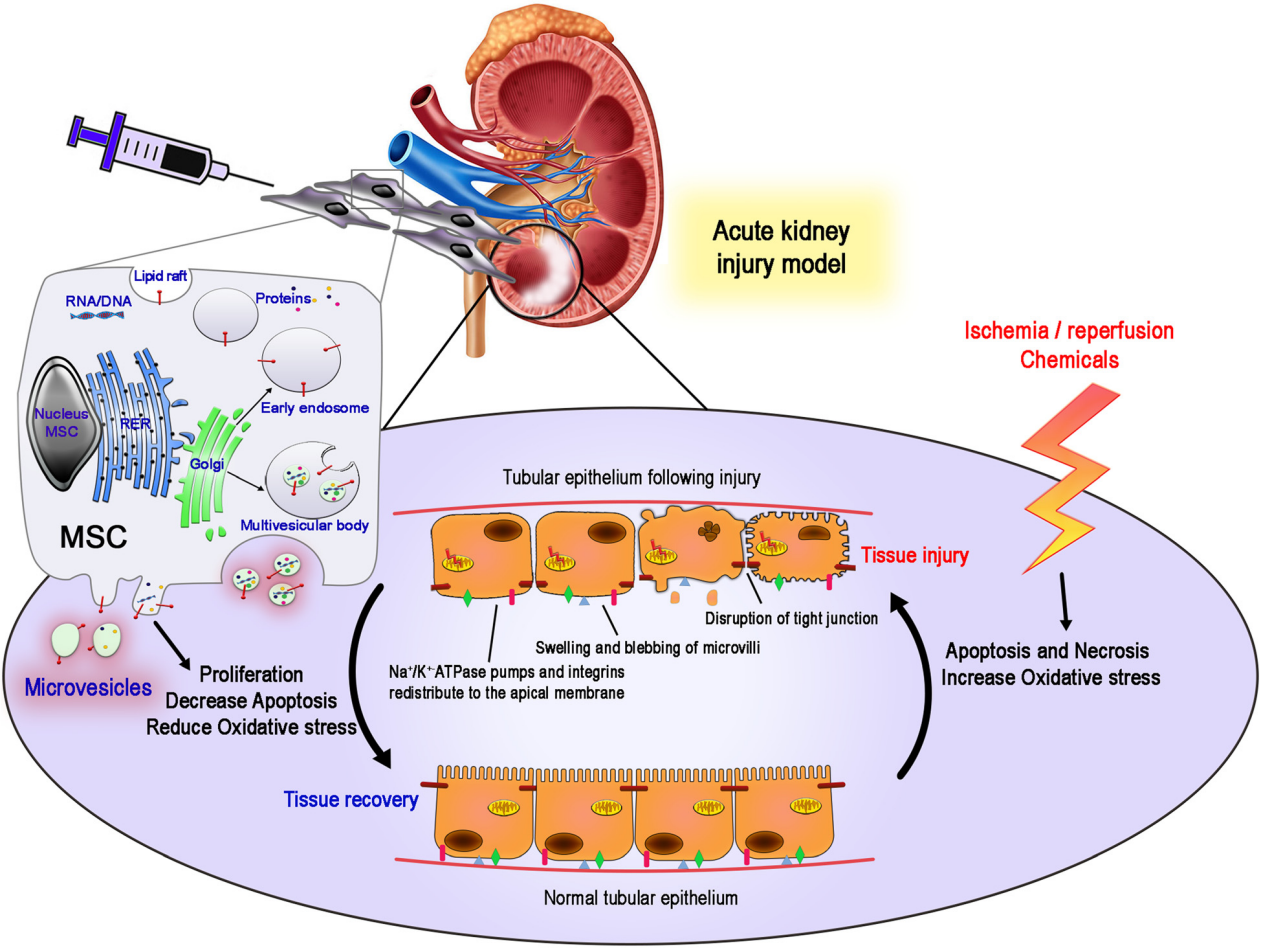
The therapeutic potential of MSC microvesicles in kidney regeneration. MSC MV mediate anti-apoptotic and pro-proliferative effects, simultaneously reducing oxidative stress to stimulate renal regeneration after acute kidney injury

In a cisplatin-induced mouse model of AKI, Morigi et al. showed that UC-MSC could stimulate endogenous target cells to produce regenerative factors, including a robust HGF expression enhanced by hypoxic conditions and inflammatory cytokines [148]. Moreover, Tögel et al. suggested that MSC exert their renal protection through inhibition of pro-inflammatory cytokines [139]. These reparative roles of MSC are likely to be multifactorial and include the provision of cytokines to limit apoptosis, enhance proliferation, and dampen the inflammatory response [145]. This hypothesis has been supported by a gene therapy approach, in which modified MSC expressing tissue kallikrein generated a benefit to tubular injury thanks to regeneration and anti-inflammatory action [149]. Additional gene therapy strategies for kidney repair are reported in Additional file 6: Link 6.2. Besides the mentioned humoral factors, the role of MV secretion in MSC therapy has recently been outlined (Fig. 3). It has been demonstrated that MV released from MSC mimic their beneficial effects for the treatment of a glycerol-induced model of AKI and ischemia–reperfusion injury [150, 151]. The same group described that MV delivery may retain similar efficacy as human BM-MSC injections. Recent reports began deciphering the molecular pathways modulated by MSC-MV in the context of renal regeneration. Specifically, MSC-MV induced the expression of several anti-apoptotic genes, including Bcl-XL, Bcl2, and baculoviral IAP repeat containing 8, in renal tubular epithelial cells while simultaneously downregulating pro-apoptotic genes such as caspase 1, caspase 8, and lymphotoxin-alpha [152].

Thus, MSC-MV may confer an anti-apoptotic phenotype necessary for tissue repair. In addition, MSC-MV stimulate renal cell proliferation by inducing the phosphorylation and subsequent activation of ERK 1/2, and blockade of ERK activation with a chemical inhibitor significantly reduces cell proliferation after MSC-MV treatment [153]. Although the exact molecules in the MV that mediate the anti-apoptotic and pro-proliferative effects have not been identified, these data demonstrate the ability of MSC-MV to simultaneously modulate several different pathways to stimulate renal regeneration. To date and to the best of our knowledge, no clinical study on microvesicles and AKI has been reported.

## The skeletal tissues

Failure of bone repair is often associated with a relevant morbidity. Therapies using recombinant BMP with or without biomaterials show promise of becoming a clinically relevant procedure. However, the lack of optimal matrices for controlled, sustained BMP delivery, a short biological half-life of BMP, and the absence of appropriate BMP responsive cells in the fracture environment limit their usefulness [154]. To overcome these limitations, bone engineering methods using MSC and scaffolds provide promising new approaches for bone repair (Table 1; Additional file 7: Link 7.1) [155]. MSC, as key progenitor cells for bone regeneration, have been historically investigated to repair skeletal tissues [156–158] alone or in combination with osteoinductive factors, such as BMP-2 [159, 160]. Pre-clinical and clinical investigations have successfully combined BMP-2 with MSC as therapy for bone defects [161] and several vectors have been tested to provide these trophic factors to skeleton (Table 1; Additional file 7: Link 7.2). In particular, BMP were delivered as liposome-mediated plasmid DNA, adenoviral vectors, and lentiviral vectors [155]. These BMP-2-modified MSC increase alkaline phosphatase activity, mineralization, and cell proliferation, and induce ectopic bone formation, heal critical size bone defects, and repair fracture triggering spinal fusion in vivo [162]. In a recent publication, we revealed that the osteogenic performance of BM-MSC can be empowered by gene modification that introduces Homeobox protein Hox-B7, which in turn promotes an autocrine loop of bFGF—a key player in proliferation and osteogenic differentiation [163]. Beside a direct differentiation into bone cells, Otsuru et al. showed that MSC stimulate bone growth in a model of osteogenesis imperfecta by secreting soluble mediators, ultimately resulting in growth-plate chondrocyte proliferation leading to bone elongation [164].

The historical heritage that MSC retain on bone regeneration represents an advantage in their clinical implementation versus other target tissues. Several clinical studies are ongoing for non-union bone defects, mandible regeneration, osteonecrosis, osteogenesis imperfecta, and vertebral regeneration [165] (www.ClinicalTrials.gov). While these challenging trials are still revealing uncertainties for MSC as a cure for bone defects in humans, they are clearly indicating a path for the development of MSC-based therapeutics on a large scale, with a solid benefit in defined clinical indications [166].

With regards to cartilage, chondrocytes have a limited regenerative potential [167], most likely because of their avascularity and a low cellularity. Although current surgical therapeutic procedures for cartilage repair are clinically useful, they cannot restore a normal articular surface, in particular where inflammatory conditions exist. To overcome these drawbacks, MSC are being considered for their ability to differentiate into cartilage and act as immunosuppressive and anti-inflammatory agents in a variety of cartilage diseases. Among them, Augello et al. reported that a single intraperitoneal injection of allogeneic MSC was sufficient to prevent the occurrence of cartilage erosion in immunized mice, suggesting that MSC might act by inhibiting the activation and proliferation of tissue-specific autoreactive T cell clones, probably by educating antigen-specific Tregs [168].

Similarly, joint destruction caused by persistent inflammation, such as in rheumatoid arthritis (RA), is a possible clinical target for cartilage repair using BM-MSC. A number of studies, based mainly on experimental animal models, have recently provided interesting data on the potential of BM-MSC to suppress local inflammation and tissue damage in RA [169]. Other studies ascribe the significant reduction in the severity of arthritis to the ability of MSC to promote the downregulation of pro-inflammatory cytokines such as TNF-α, IL-1, and interferon-γ and the concomitant upregulation of IL-10 [170, 171].

Based on these in vitro and in vivo evidences, MSC have been introduced in humans for experimental purposes within trials investigating safety and efficacy [10, 12, 72, 172]. Outlining the safety of the approach, the intra-articular delivery of MSC appears promising although still requires additional investigation to definitively ameliorate the chondrogenic actions of MSC [173–175].

Current research offers a growing number of bioactive reagents, including proteins and nucleic acids, that may be used to augment different aspects of the repair process. It is difficult to effectively deliver these agents and gene transfer approaches are being developed to provide their sustained synthesis at sites of damage by MSC delivery. The list of potentially useful cDNAs for cartilage repair comprises members of the TGF-β superfamily, several BMPs, IGF-1, FGF, and EGF. Experimental data generated so far have shown that genetically modified MSC allow sustained transgene expression when transplanted into articular cartilage defects in vivo, and enhance the structural features of cartilaginous tissue repair [176, 177].

## Conclusions

This review highlights that injured organs may benefit from MSC as regenerative tools able to differentiate accordingly, secrete useful factors, or both, with the final effect of counteracting damages. The reported data generally indicate amelioration, while less frequently clarifications on mechanism(s) driving the observed therapeutic potential have been addressed. Technicalities limit current understanding; nevertheless, efforts are being made to transfer knowledge from the laboratory to the clinic and vice versa to identify the drivers of the observed MSC impacts. Studies will be implemented on deciphering the ideal MSC tissue source for precise clinical application, as well as on the best delivery manner to exploit MSC potential through better cellular retention and optimized recruitment. More information will be gathered on the still poorly explored potential of MSC, such as whether MV, previously considered to be cell debris, may become an important mediator of intercellular communication. Collectively, this research will contribute to better characterized MSC that can be selected by physicians based on their patient needs, and on more precise information on the pivotal properties of MSC that lead to their enduring benefits.

## Additional files

Additional file 1:
**Link 1.1** Overview of the main experimental findings on the impact of wild-type MSC in diseases associated with the central and peripheral nervous system. **Link 1.2** Overview of the main experimental findings on the impact of gene-modified MSC in diseases associated with the central and peripheral nervous system. (DOCX 34 kb) Additional file 2:
**Link 2.1** Overview of the main pre-clinical findings on the impact of wild-type MSC in heart diseases. **Link 2.2** Overview of the main pre-clinical findings on the impact of gene-modified MSC in heart diseases. (DOCX 39 kb) Additional file 3:
**Link 3.1** Overview of the main pre-clinical findings on the impact of wild-type MSC in lung diseases. **Link 3.2** Overview of the main pre-clinical findings on the impact of gene-modified MSC in lung diseases. (DOCX 24 kb) Additional file 4:
**Link 4.1** Overview of the main pre-clinical findings on the impact of wild-type MSC in liver diseases. **Link 4.2** Overview of the main pre-clinical findings on the impact of gene-modified MSC in liver diseases. (DOCX 22 kb) Additional file 5:
**Link 5.1** Overview of the main pre-clinical findings on the impact of wild-type MSC in pancreatic diseases. **Link 5.2** Overview of the main pre-clinical findings on the impact of gene-modified MSC in pancreatic diseases. (DOCX 20 kb) Additional file 6:
**Link 6.1** Overview of the main pre-clinical findings on the impact of wild-type MSC in renal diseases. **Link 6.2** Overview of the main pre-clinical findings on the impact of gene-modified MSC in renal diseases. (DOCX 29 kb) Additional file 7:
**Link 7.1** Overview of the main pre-clinical findings on the impact of wild-type MSC in skeletal diseases. **Link 7.2** Overview of the main pre-clinical findings on the impact of gene-modified MSC in skeletal diseases. (DOCX 24 kb)

## Abbreviations

AD: adipose
ADK: adenosine kinase
AKI: acute kidney injury
ALS: amyotrophic lateral sclerosis
Ang: angiopoietin
AP: acute pancreatitis
Bcl: B-cell lymphoma
BDNF: brain-derived neurotrophic factor
bFGF: basic fibroblast growth factor
BM: bone marrow
BMP: bone morphogenetic protein
BPD: bronchopulmonary dysplasia
CXCR4: C-X-C chemokine receptor type 4
EGF: epidermal growth factor
EPO: erythropoietin
ERK 1/2: extracellular signal-regulated kinase 1/2
GAD: glutamic acid decarboxylase
GDNF: glial cell-derived neurotrophic factor
HD: Huntington’s disease
HGF: hepatocyte growth factor
IGF: insulin-like growth factor
IL: interleukin
i.v.: intravenous
MI: myocardial infarction
MIP: macrophage inflammatory protein
MMP: matrix metalloproteinases
MS: multiple sclerosis
MSC: multipotent mesenchymal stem/stromal cells
MV: microvesicles
NT: neurotrophin
PBMC: peripheral blood mononuclear cells
PD: Parkinson’s disease
PDGF: platelet-derived growth factor
RA: rheumatoid arthritis
SCI: spinal cord injury
SDF: stromal cell-derived factor
STZ: streptozotocin
T1D: type 1 diabetes
T2D: type 2 diabetes
TGF: transforming growth factor
TNF: tumor necrosis factor
hUC: human umbilical cord
VEGF: vascular endothelial growth factor
