# Supplementary material for: Mesenchymal stem/stromal cells as a delivery platform in cell and gene therapies
Source: BMC Med. 2015 Aug 12;13:186. doi: 10.1186/s12916-015-0426-0 (PMC4534031; doi:10.1186/s12916-015-0426-0)
Supplement: Additional file 1: — Link 1.1 Overview of the main experimental findings on the impact of wild-type MSC in diseases associated with the central and peripheral nervous system. Link 1.2 Overview of the main experimental findings on the impact of gene-modified MSC in diseases associated with the central and peripheral nervous system. (DOCX 34 kb) [file 12916_2015_426_MOESM1_ESM.docx]

**Link 1.1 Overview of the main experimental findings on the impact of wild type MSC in diseases associated with central and peripheral nervous system**

| **DISEASE (MODEL)** | **MSC SOURCE** | **TYPE OF STUDY** | **ROUTE OF ADMINISTRATION** | **PROPOSED MECHANISM** | **REF** |
| --- | --- | --- | --- | --- | --- |
| Stroke  (middle cerebral artery occlusion) | Rat BM | In vivo (rat) | Tail vein | Promoted neurovascular remodeling, functional recovery | [1] |
| Amyotrophic lateral sclerosis  (SOD-1^G93A^ mutant mouse) | Murine AD | In vivo (mouse) | Tail vein | Influence on astrocytic secretome. Up regulation of GDNF and bFGF | [2] |
| Stroke  (middle cerebral artery occlusion) | Rat BM | In vivo (rat) and in vitro | Tail vein | Transfer of miR-133b to neural cells via exosomes improves neurite outgrowth | [3] |
| Glutamate excitotoxicity  (kainic acid-induced) | Murine BM | In vivo (mouse) and in vitro | N/A | Reduce neuronal sensitivity to glutamate | [4] |
| Huntington’s disease  (quinolinic acid-induced and R6/2-J2 mouse) | Human BM | In vivo (mouse) | Tail vein | Low rate of differentiation, improved angiogenesis, neuroprotection | [5] |
| Krabbe’s disease  (twitcher mouse) | Murine AD and BM | In vivo (mouse) | Bilateral intraventricular | Secretion of functional GALC enzyme, low rate of differentiation. Immunosuppressive effect | [6] |
| Cerebral ischemia  (cerebral artery occlusion model) | B10 human MSC line | In vivo (rat) | Jugular vein | Paracrine effect | [7] |
| Spinal cord injury  (spinal contusion) | Rat BM | In vivo (rat) | Intraspinal | Secretion of neurotrophic factors (BDNF, GDNF) | [8] |
| Multiple sclerosis  (chronic autoimmune encephalomyelitis) | Murine AD and BM | In vivo (mouse) | Tail vein | Systemic immune modulation on autoreactive T cells. Induction of local neurogenesis through secretion of neural growth factors | [9] |
| Spinal cord injury  (complete transection) | Human UC | In vivo (rat) | Intraspinal | Secretion of growth factors (NAP-2, NT-3, VEGFR-3) | [10] |
| Parkinson’s disease  (6-OHDA-induced) | Rat AD | In vivo (rat) | Ipsilateral to striatum | Secretion of neurotrophic factors | [11] |
| Amyotrophic lateral sclerosis  (SOD-1^G93A^ mutant mouse) | Human BM | In vivo (transgenic mouse) | Intraspinal | Production of trophic factors (VEGF, BDNF) and supply of wild type SOD | [12] |
| Multiple sclerosis  (chronic autoimmune encephalomyelitis) | Murine BM | In vivo (mouse) | Intravenous | Inhibition of B-cells infiltration in CNS. Decrease of in-vivo production of pathogenic antibodies | [13] |

**Link 1.2 Overview of the main experimental findings on the impact of gene modified MSC in diseases associated with central and peripheral nervous system**

| **DISEASE (MODEL)** | **MSC SOURCE** | **VECTOR** | **GENE** | **TYPE OF STUDY** | **ROUTE OF ADMINISTRATION** | **PROPOSED MECHANISM** | **REF** |
| --- | --- | --- | --- | --- | --- | --- | --- |
| Parkinson’s disease  (MPTP-induced) | Monkey BM | Lentiviral | Human GDNF | In vivo (monkey) | Intracerebral | Functional improvement | [14] |
| Parkinson’s disease  (rotenone-induced) | Human UC | AAV | Human VEGF | In vivo (rat) | Intrastriatal | Differentiation, increased VEGF secretion, decreased loss of neurons, improvement | [15] |
| Parkinson’s disease  (6-OHDA-induced) | Rat BM | Lentiviral | TH, GDNF | In vivo (rat) | Intrastriatal | High secretion, functional improvement | [16] |
| Parkinson’s disease  (6-OHDA-induced) | Human BM | Lentiviral | Human GDNF | In vivo (rat) | Intrastriatal | Possible release of trophic factors, rejuvenation of DA fibers in vivo, recovery of DA neurons, GDNF secretion increased, functional improvement | [17] |
| Parkinson’s disease  (6-OHDA-induced) | Rat BM | Retroviral | Human GDNF | In vivo (rat) | Intrastriatal | Local trophic effect, increased GDNF secretion, neuronal differentiation | [18] |
| Parkinson’s disease  (6-OHDA-induced) | Rat BM | Lentiviral | GDNF | In vivo (rat) | Intrastriatal | Neuron rescue from neurotoxicity, behavioral improvement | [19] |
| Stroke  (middle cerebral artery occlusion) | Rat BM | Lentiviral | Rat CXCR4 | In vivo (rat) | Femoral vein | Enhanced mobilization, promotes cell repair and angiogenesis, reduced infarct volume, functional neurological recovery | [20] |
| Alzheimer’s Disease)  (transgenic mouse) | Human BM | Non-viral | GLP-1 | In vivo (mouse) | Intraventricular | Decreased amyloid depositions or suppression of glial and microglial responses | [21] |
| Brain Ischemia  (carotid artery occlusion) | Human Neural | Lentiviral | Human Galectin-1 | In vivo (Mongolian gerbils) | Caudate nucleus of lesion hemisphere | Reduced infarct volume, preservation of host-tissue by release of trophic factors, overexpression could increase neurite outgrowth, functional recovery | [22] |
| Spinal cord injury  (Twitcher mouse) | Human neural, Mouse neural | Lentiviral | Murine GAL-C | In vivo (mouse) | Intracerebroventricular | Enzyme secretion and distribution, modulation of inflammation, neuroprotection, cell replacement | [23] |
| Huntington disease  (YAC 128 mouse) | Mouse BM | Non-viral | BDNF, NGF | In vivo (mouse) | Bilaterally into striatum | Protection, preserved motor function, neurodegenerative process delayed | [24] |
| Spinal cord injury  (spinal contusion) | Human neural | Lentiviral | Human Galectin-1 | In vivo (marmosets) | Lesion site | Autocrine/paracrine mechanisms, secretion, differentiation, survival, regenerative process | [25] |
| Spinal cord injury  (complete transection) | Rat BM | Adenoviral | Human NT-3 | In vivo (rat) | Lesion site | Neuronal differentiation, increased NT-3 secretion, functional and structural improvement, axonal regeneration, increased neuronal survival | [26] |
| Stroke  (middle cerebral artery occlusion) | Human BM | Fiber mutant adenoviral | Human Ang-1,  human VEGF | In vivo (rat) | Intravenous | Stimulated endogenous repair, reduced infarct volume, increased angiogenesis, structural-functional recovery | [27] |
| Epilepsy  (kainic acid-induced) | Human ES | Lentiviral | microRNA against ADK | In vivo (mouse) | Infrahippocampal cleft | Reduction in seizures and neuronal loss, neuroprotection | [28] |
| Multiple sclerosis (experimental allergic encephalomyelitis) | Human | Adenoviral | Human CNTF | In vivo (mouse) | Intravenous | Increased secretion, neuronal functional recovery, disease onset delayed, immunoregulatory activity, inhibiting inflammation, homing, reducing demyelination and stimulating oligodendrogenesis | [29] |
| Multiple sclerosis (experimental allergic encephalomyelitis) | Adult neural | Lentiviral | IL-10 | In vivo (mouse) | Intravenous and intracerebroventricular | Suppress autoimmune function and reduced myelin damage, differentiation to promote exogenous remyelination, reduce local inflammation to promote endogenous remyelination | [30] |
| Parkinson’s disease  (6-OHDA-induced) | Rat and human | Non-viral | Murine NICD | In vivo (rat) | Ipsilateral to striatum | Possible release of trophic factors, neuronal characteristics, rejuvenation of DA fibers in vivo, recovery of DA neurons, GDNF secretion increased, functional improvement | [31] |

**Abbreviations:** 6-OHDA: 6-hydroxydopamine; AAV: Adeno-associated virus; AD: Adipose; ADK: Adenosine kinase; Ang-1: Angiopoietin 1; BDNF: Brain-derived neurotrophic factor; bFGF: Basic fibroblast growth factor; BM: Bone marrow; CNS: Central nervous system; CNTF: Ciliary neurotrophic factor; CXCR4; C-X-C chemokine receptor type 4; DA: Dopaminergic; GALC: Galactosylceramidase; GDNF: Glial cell-derived neurotrophic factor; GLP-1: Glucagon-like peptide-1; IL-10: Interleukin-10; MPTP: 1-methyl-4-phenyl-1,2,3,6-tetrahydropyridine; NAP-2: Neutrophil-activating protein-2; NGF: Nerve growth factor; NICD: Notch intracellular domain; NT-3: Neurotrophin-3; UC: Umbilical cord; SOD-1: Superoxide dismutase-1; TH: Tyrosine hydroxylase; VEGF: Vascular endothelial growth factor; VEGFR-3: Vascular endothelial growth factor receptor-3.

**RELATED REFERENCES**

1. Xin H, Li Y, Cui Y, Yang JJ, Zhang ZG, Chopp M: **Systemic administration of exosomes released from mesenchymal stromal cells promote functional recovery and neurovascular plasticity after stroke in rats**. *J Cereb Blood Flow Metab* 2013, **33**:1711–1715.

2. Marconi S, Bonaconsa M, Scambi I, Squintani GM, Rui W, Turano E, Ungaro D, D’Agostino S, Barbieri F, Angiari S, Farinazzo A, Constantin G, Del Carro U, Bonetti B, Mariotti R: **Systemic treatment with adipose-derived mesenchymal stem cells ameliorates clinical and pathological features in the amyotrophic lateral sclerosis murine model**. *Neuroscience* 2013, **248C**:333–343.

3. Xin H, Li Y, Buller B, Katakowski M, Zhang Y, Wang X, Shang X, Zhang ZG, Chopp M: **Exosome-Mediated Transfer of miR-133b from Multipotent Mesenchymal Stromal Cells to Neural Cells Contributes to Neurite Outgrowth**. *STEM CELLS* 2012, **30**:1556–1564.

4. Voulgari-Kokota A, Fairless R, Karamita M, Kyrargyri V, Tseveleki V, Evangelidou M, Delorme B, Charbord P, Diem R, Probert L: **Mesenchymal stem cells protect CNS neurons against glutamate excitotoxicity by inhibiting glutamate receptor expression and function**. *Exp Neurol* 2012, **236**:161–170.

5. Lin Y-T, Chern Y, Shen C-KJ, Wen H-L, Chang Y-C, Li H, Cheng T-H, Hsieh-Li HM: **Human Mesenchymal Stem Cells Prolong Survival and Ameliorate Motor Deficit through Trophic Support in Huntington’s Disease Mouse Models**. *PLoS ONE* 2011, **6**:1–17.

6. Ripoll CB, Flaat M, Klopf-Eiermann J, Fisher-Perkins JM, Trygg CB, Scruggs BA, McCants ML, Leonard HP, Lin AF, Zhang S, Eagle ME, Alvarez X, Li YT, Li SC, Gimble JM, Bunnell BA: **Mesenchymal lineage stem cells have pronounced anti-inflammatory effects in the twitcher mouse model of Krabbe’s disease**. *Stem Cells Dayt Ohio* 2011, **29**:67–77.

7. Wakabayashi K, Nagai A, Sheikh AM, Shiota Y, Narantuya D, Watanabe T, Masuda J, Kobayashi S, Kim SU, Yamaguchi S: **Transplantation of human mesenchymal stem cells promotes functional improvement and increased expression of neurotrophic factors in a rat focal cerebral ischemia model**. *J Neurosci Res* 2010, **88**:1017–1025.

8. Gu W, Zhang F, Xue Q, Ma Z, Lu P, Yu B: **Transplantation of bone marrow mesenchymal stem cells reduces lesion volume and induces axonal regrowth of injured spinal cord**. *Neuropathology* 2010, **30**:205–217.

9. Constantin G, Marconi S, Rossi B, Angiari S, Calderan L, Anghileri E, Gini B, Dorothea Bach S, Martinello M, Bifari F, Galiè M, Turano E, Budui S, Sbarbati A, Krampera M, Bonetti B: **Adipose-Derived Mesenchymal Stem Cells Ameliorate Chronic Experimental Autoimmune Encephalomyelitis**. *Stem Cells* 2009, **27**:2624–2635.

10. Yang C-C, Shih Y-H, Ko M-H, Hsu S-Y, Cheng H, Fu Y-S: **Transplantation of Human Umbilical Mesenchymal Stem Cells from Wharton’s Jelly after Complete Transection of the Rat Spinal Cord**. *PLoS ONE* 2008, **3**:e3336.

11. McCoy MK, Martinez TN, Ruhn KA, Wrage PC, Keefer EW, Botterman BR, Tansey KE, Tansey MG: **Autologous transplants of Adipose-Derived Adult Stromal (ADAS) cells afford dopaminergic neuroprotection in a model of Parkinson’s disease**. *Exp Neurol* 2008, **210**:14–29.

12. Vercelli A, Mereuta OM, Garbossa D, Muraca G, Mareschi K, Rustichelli D, Ferrero I, Mazzini L, Madon E, Fagioli F: **Human mesenchymal stem cell transplantation extends survival, improves motor performance and decreases neuroinflammation in mouse model of amyotrophic lateral sclerosis**. *Neurobiol Dis* 2008, **31**:395–405.

13. Gerdoni E, Gallo B, Casazza S, Musio S, Bonanni I, Pedemonte E, Mantegazza R, Frassoni F, Mancardi G, Pedotti R, Uccelli A: **Mesenchymal stem cells effectively modulate pathogenic immune response in experimental autoimmune encephalomyelitis**. *Ann Neurol* 2007, **61**:219–227.

14. Ren Z, Wang J, Wang S, Zou C, Li X, Guan Y, Chen Z, Zhang YA: **Autologous transplantation of GDNF-expressing mesenchymal stem cells protects against MPTP-induced damage in cynomolgus monkeys**. *Sci Rep* 2013, **3**.

15. Xiong N, Zhang Z, Huang J, Chen C, Zhang Z, Jia M, Xiong J, Liu X, Wang F, Cao X, Liang Z, Sun S, Lin Z, Wang T: **VEGF-expressing human umbilical cord mesenchymal stem cells, an improved therapy strategy for Parkinson’s disease**. *Gene Ther* 2011, **18**:394–402.

16. Shi D, Chen G, Lv L, Li L, Wei D, Gu P, Gao J, Miao Y, Hu W: **The effect of lentivirus-mediated TH and GDNF genetic engineering mesenchymal stem cells on Parkinson’s disease rat model**. *Neurol Sci Off J Ital Neurol Soc Ital Soc Clin Neurophysiol* 2011, **32**:41–51.

17. Glavaski-Joksimovic A, Virag T, Mangatu TA, McGrogan M, Wang XS, Bohn MC: **Glial cell line-derived neurotrophic factor-secreting genetically modified human bone marrow-derived mesenchymal stem cells promote recovery in a rat model of Parkinson’s disease**. *J Neurosci Res* 2010, **88**:2669–2681.

18. Moloney TC, Rooney GE, Barry FP, Howard L, Dowd E: **Potential of rat bone marrow-derived mesenchymal stem cells as vehicles for delivery of neurotrophins to the Parkinsonian rat brain**. *Brain Res* 2010, **1359**:33–43.

19. Wu J, Yu W, Chen Y, Su Y, Ding Z, Ren H, Jiang Y, Wang J: **Intrastriatal transplantation of GDNF-engineered BMSCs and its neuroprotection in lactacystin-induced Parkinsonian rat model**. *Neurochem Res* 2010, **35**:495–502.

20. Yu X, Chen D, Zhang Y, Wu X, Huang Z, Zhou H, Zhang Y, Zhang Z: **Overexpression of CXCR4 in mesenchymal stem cells promotes migration, neuroprotection and angiogenesis in a rat model of stroke**. *J Neurol Sci* 2012, **316**:141–149.

21. Klinge PM, Harmening K, Miller MC, Heile A, Wallrapp C, Geigle P, Brinker T: **Encapsulated native and glucagon-like peptide-1 transfected human mesenchymal stem cells in a transgenic mouse model of Alzheimer’s disease**. *Neurosci Lett* 2011, **497**:6–10.

22. Yamane J, Ishibashi S, Sakaguchi M, Kuroiwa T, Kanemura Y, Nakamura M, Miyoshi H, Sawamoto K, Toyama Y, Mizusawa H, others: **Transplantation of human neural stem/progenitor cells overexpressing galectin-1 improves functional recovery from focal brain ischemia in the mongolian gerbil**. *Mol Brain* 2011, **4**:35.

23. Neri M, Ricca A, di Girolamo I, Alcala’-Franco B, Cavazzin C, Orlacchio A, Martino S, Naldini L, Gritti A: **Neural Stem Cell Gene Therapy Ameliorates Pathology and Function in a Mouse Model of Globoid Cell Leukodystrophy**. *STEM CELLS* 2011, **29**:1559–1571.

24. Dey ND, Bombard MC, Roland BP, Davidson S, Lu M, Rossignol J, Sandstrom MI, Skeel RL, Lescaudron L, Dunbar GL: **Genetically engineered mesenchymal stem cells reduce behavioral deficits in the YAC 128 mouse model of Huntington’s disease**. *Behav Brain Res* 2010, **214**:193–200.

25. Yamane J, Nakamura M, Iwanami A, Sakaguchi M, Katoh H, Yamada M, Momoshima S, Miyao S, Ishii K, Tamaoki N, Nomura T, Okano HJ, Kanemura Y, Toyama Y, Okano H: **Transplantation of galectin-1-expressing human neural stem cells into the injured spinal cord of adult common marmosets**. *J Neurosci Res* 2010:NA–NA.

26. Zhang W, Yan Q, Zeng Y-S, Zhang X-B, Xiong Y, Wang J-M, Chen S-J, Li Y, Bruce IC, Wu W: **Implantation of adult bone marrow-derived mesenchymal stem cells transfected with the neurotrophin-3 gene and pretreated with retinoic acid in completely transected spinal cord**. *Brain Res* 2010, **1359**:256–271.

27. Toyama K, Honmou O, Harada K, Suzuki J, Houkin K, Hamada H, Kocsis JD: **Therapeutic benefits of angiogenetic gene-modified human mesenchymal stem cells after cerebral ischemia**. *Exp Neurol* 2009, **216**:47–55.

28. Boison D: **Engineered adenosine-releasing cells for epilepsy therapy: human mesenchymal stem cells and human embryonic stem cells**. *Neurother J Am Soc Exp Neurother* 2009, **6**:278–283.

29. Lu Z, Hu X, Zhu C, Wang D, Zheng X, Liu Q: **Overexpression of CNTF in Mesenchymal Stem Cells reduces demyelination and induces clinical recovery in experimental autoimmune encephalomyelitis mice**. *J Neuroimmunol* 2009, **206**:58–69.

30. Yang J, Jiang Z, Fitzgerald DC, Ma C, Yu S, Li H, Zhao Z, Li Y, Ciric B, Curtis M, Rostami A, Zhang G-X: **Adult neural stem cells expressing IL-10 confer potent immunomodulation and remyelination in experimental autoimmune encephalitis**. *J Clin Invest* 2009, **119**:3678–3691.

31. Dezawa M, Kanno H, Hoshino M, Cho H, Matsumoto N, Itokazu Y, Tajima N, Yamada H, Sawada H, Ishikawa H, Mimura T, Kitada M, Suzuki Y, Ide C: **Specific induction of neuronal cells from bone marrow stromal cells and application for autologous transplantation**. *J Clin Invest* 2004, **113**:1701–1710.
