# Supplementary material for: Mesenchymal stem/stromal cells as a delivery platform in cell and gene therapies
Source: BMC Med. 2015 Aug 12;13:186. doi: 10.1186/s12916-015-0426-0 (PMC4534031; doi:10.1186/s12916-015-0426-0)
Supplement: Additional file 2: — Link 2.1 Overview of the main pre-clinical findings on the impact of wild-type MSC in heart diseases. Link 2.2 Overview of the main pre-clinical findings on the impact of gene-modified MSC in heart diseases. (DOCX 39 kb) [file 12916_2015_426_MOESM2_ESM.docx]

**Link 2.1 Overview of the main pre-clinical findings on the impact of wild type MSC in heart diseases**

| **DISEASE (MODEL)** | **MSC SOURCE** | **TYPE OF STUDY** | **ROUTE OF ADMINISTRATION** | **PROPOSED MECHANISM** | **REF** |
| --- | --- | --- | --- | --- | --- |
| Myocardial infarction  (ischaemia-reperfusion) | Rat BM and AD | In vivo (rat) | Intramyocardial | N/A | [1] |
| Myocardial infarction  (coronal artery ligation) | Human Endometrium | In vivo (rat) | Intramyocardial | Activation of AKT, ERK1/2 and STAT3 and inhibition of the p38 signalling pathway, decreased apoptosis and promotion of cell proliferation through TGF-β2 and EGF production, C-kit+ cell recruitment | [2] |
| Diabetic cardiac autonomic neuropathy (STZ-induced) | Rat BM | In vivo (rat) | Intramyocardial | Secretion of NT-3 and NGF | [3] |
| Acute myocardial infarction  (coronal artery ligation) | Human AD | In vivo (mouse) | Intramyocardial | Differentiation in vascular and myocardial cells, secretion of pro-angiogenic factors, apoptosis reduction via secretion of IGF-1 | [4] |
| Myocardial Infarction (ischemia and reperfusion injury) | Human ESC | In vivo (mouse) | Intracoronary | Reduced injury | [5] |
| Myocardial infarction  (ischaemia-reperfusion) | Swine BM | In vivo (pig) | Transendocardial | Direct differentiation or cell fusion and stimulation of endogenous cardiomyocyte turnover | [6] |
| Acute myocardial infarction  (coronal artery ligation) | Human AD | In vivo (rat) | Intramyocardial | Promote angiogenesis and cardiac nerve sprouting through grow factors secretion | [7] |
| Acute myocardial infarction  (coronal artery ligation) | Rat AD | In vivo (rat) | Intraventricular | Potent VEGF-mediated pro-angiogenic effect | [8] |
| Acute myocardial infarction  (coronal artery ligation) | Rat AD | In vivo (rat) | Intramyocardial | Differentiation into vascular endothelial cells and production of VEGF | [9] |
| Myocardial Infarction (ischemia and reperfusion injury) | Human ESC | In vivo (pig) | Intracoronary | Reduced infarct size | [10] |
| Acute myocardial infarction  (coronal artery ligation) | Murine BM | In vivo (mouse) | Intraventricular | Reduction of apoptotic cardiomyocytes in periinfarct area by upregulation of AKT and increased synthesis of inducible nitric oxide synthase | [11] |
| Acute myocardial infarction  (coronal artery ligation) | Rat AD | In vivo (rat) | Transplant of monolayered MSC | Low rate of differentiation and pro-angiogenic cytokine production | [12] |
| Chronic myocardial ischemia (coronal artery constriction) | Canine BM | In vivo (dog) | Intramyocardial | Enhance angiogenesis by transdifferentiation into smooth muscle cells and by paracrine effect | [13] |
| Acute myocardial infarction  (coronal artery ligation) | Rat BM | In vivo (rat) | Jugular vein | Enhancement of angiogenesis and myogenesis | [14] |

**Link 2.2 Overview of the main pre-clinical findings on the impact of gene modified MSC in heart diseases**

| **DISEASE (MODEL)** | **MSC SOURCE** | **VECTOR** | **GENE** | **TYPE OF STUDY** | **ROUTE OF ADMINISTRATION** | **PROPOSED MECHANISM** | **REF** |
| --- | --- | --- | --- | --- | --- | --- | --- |
| Myocardial Infarction (coronary artery ligation) | Murine BM | Adenoviral | Human PEDF | In vivo (mouse) | Tail vein | Induced cellular profile changes, regulated proliferation and migration, paracrine actions, improved therapeutic efficacy | [15] |
| Myocardial Infarction (coronary artery ligation) | Porcine BM | Adenoviral | Human VEGF/HGF | In vivo (pig) | Coronary vein | Improved cardiac function and perfusion, increased cell survival and neovascularization, reduced fibrosis | [16] |
| Myocardial Infarction (coronary artery ligation) | Human ESC | Lentiviral | Human HO-1 | In vivo (rat) | Directly into scar | Increased expression of pro-survival and angiogenesis-promoting genes, cell-to-cell communication | [17] |
| Myocardial Infarction (coronary artery ligation) | Old Human BM | Non-viral | TIMP-3/VEGF | In vivo (rat) | One site in the center of the ischemic myocardium and into three sites in the border of the infarct region | Differentiation in vitro, increased  survival of the transplanted old hMSCs, reduced infarct size, restored cardiac function, increased angiogenesis, paracrine mechanism, secretion of cytokines, modulated matric remodeling | [18] |
| Acute Myocardial Infarction (coronary artery ligation) | Rat BM | Non-viral + nanoparticles | Hypoxamir-210 | In vivo (rat) | Intramyocardially at multiple sites in and around the infarct zone | Cytoprotective effect, promoted survival, preserved ventricular function | [19] |
| Myocardial Infarction (coronary artery ligation) | Rat BM | Lentiviral | Periostin | In vivo (rat) | Tissues from the injured region to the border | Prevented apoptosis, protective, enhanced survival, functional recovery | [20] |
| Myocardial Infarction (coronary artery ligation) | Rat BM | Non-viral | Human VEGF | In vivo (rat) | Anterior and lateral aspects of the contracting wall bordering the infarct | Increased neovascularization, reduced apoptotic cells | [21] |
| Myocardial Infarction (coronary artery ligation) | Murine BM | Retroviral | Mouse Csx/Nkx2.5 and GATA-4 | In vivo (mouse) | Multiple sites in border of infarcted myocardium | Improved heart function, cytoprotective effects, improved survival, resisted apoptosis and necrosis, induced angiogenesis, promoted differentiation and improved transplantation | [22] |
| Acute Myocardial Infarction (coronary artery ligation) | Murine BM | Lentiviral | PGIS | In vivo (mouse) | Multiple points near infarct region | Anti-apoptopic and anti-inflammatory, enhanced proliferation, inhibited cytokine secretion, enhanced angiogenesis, limited cardiac remodeling, functional improvement | [23] |
| Pulmonary Hypertension (monocrotaline induced) | Rat BM | Retroviral | Prostacyclin synthase | In vivo (rat) | Tail vein | Increased mice survival, functional improvement | [24] |
| Myocardial Infarction (coronary artery ligation) | Swine BM | Lentiviral | Akt-1 | In vivo (pig) | Left anterior descending coronary artery of the infarct heart | Improved cardiac function, anti-apoptopic function, increased survival rate | [25] |
| Myocardial Infarction (coronary artery ligation) | Rat BM | Adenoviral | CXCR4 | In vivo (rat) | Peritoneum cell patch | Upregulation of chemokines, promoted angiogenesis, cardiac protection via paracrine action, LV fibrosis and function improved | [26] |
| Myocardial Infarction (coronary artery ligation) | Murine BM | Retroviral | Mouse CCR1, mouse CXCR2 | In vivo (mouse) | Intramyocardially | Higher chemotactic activity, paracrine actions, increased migration, survival and engraftment, decreased apoptosis, protective effect, prevented cardiac remodeling and restored cardiac function | [27] |
| Myocardial Infarction (coronary artery ligation) | Rat BM | Non-viral | Human HO-1 | In vivo (rat) | Multiple sites in infarct border | Anti-apoptopic, anti-oxidative capabilities, increased cell survival, secretion, cell protection, paracrine actions increased angiogenesis, improved repair | [28] |
| Acute Myocardial Infarction (coronary artery ligation) | Rat BM | Adenoviral | SDF-1, VEGF | In vivo (rat) | Multiple sites in infarct region | Improved survival, secretion, functional recovery | [29] |
| Myocardial Infarction (coronary artery ligation) | Rat BM | Lentiviral | PI3K-C2α | In vivo (rat) | Injured region to the border | Increased viability, reduced apoptosis, functional recovery | [30] |
| Acute Myocardial Infarction (coronary artery ligation) | Rat BM | AAV | TNFR | In vivo (rat) | Intramyocardial | Increased MSC survival and viability, secretion, functional improvement | [31] |
| Myocardial Infarction (coronary artery ligation) | Mouse BM | AAV | VEGF | In vivo (mouse) | Border of ischemic area | Reduced cell loss, improved cardiac function, reduced infarct size, increased survival, protective effect, paracrine actions | [32] |
| Cardiac regeneration (cardiomyopathic hamster) | Human BM | Adenoviral | VEGF | In vivo (hamsters) | Intramuscular | Secretion, trophic factors, restored cardiac function | [33] |
| Heart ischemia (chronic coronary ischaemia) | Porcine BM | AAV | Ang-1 | In vivo (pig) | Ischemic border area | Cytokine effect, functional improvement, supported neovascularization, increased tissue perfusion | [34] |
| Myocardial Infarction (coronary artery ligation) | Rat BM | Lentiviral | Integrin-linked kinase | In vivo (rat) | Anterior and lateral aspects of the contracting wall bordering the infarct | Increased cell survival and adhesion, anti-apoptotic, reduced infarct size, improved microvessel density, functional improvement | [35] |
| Acute Myocardial Infarction (coronary artery ligation) | Murine BM | Lentiviral | Mouse VEGF, mouse HGF | In vivo (mouse) | Multiple sites within the presumed infarct and border zone | Upregulated cytokine production, augmented both paracrine and autocrine mechanisms involved in cell survival and myocardial recovery, reduced apoptosis, decreased scar size | [36] |
| Myocardial Infarction (coronary artery ligation) | Rat BM | Retroviral | Akt | In vivo (rat) | Multiple sites in the border zone | Survival effect, preservation of normal metabolism, protective, production/release of paracrine factors | [37] |
| Myocardial Infarction (coronary artery ligation) | Rat BM | Lentiviral | Human Survivin | In vivo (rat) | Intramyocardial | Secretion, decreased infarct size, improved cardiac function | [38] |
| Myocardial Infarction (coronary artery ligation)) | Rat BM | Adenoviral | Hsp20 | In vivo (rat) | Anterior and lateral aspects of the contracting wall bordering the infarct | Protective, increased survival, paracrine effect by secretion of growth factors, improved cardiac remodeling and function, reduced fibrosis | [39] |
| Acute Myocardial Infarction (coronary artery ligation) | Murine BM | N/A | Human IL-8 binding protein | In vivo (rat) | Intramyocardial | Secretion, cardioprotective effects via paracrine signaling, resistant to inflammation, improved LV function | [40] |

**Abbreviations:** AAV: Adeno-associated virus; AD: Adipose; BM: Bone marrow; CCR1: C-C chemokine receptor type 1; CXCR2: C-X-C chemokine receptor type 2; CXCR4; C-X-C chemokine receptor type 4; EGF: Epidermal growth factor; ERK1/2: Extracellular signal-regulated protein kinases ½; ESC: Embryonic stem cells; HGF: Hepatocyte growth factor; HO-1: Heme oxygenase-1; Hsp20: Heat-shock protein 20; IGF-1: Insulin-like growth factor-1; IL-8: Interleukin 8; LV: Left ventricular; MSC: Mesenchymal Stem/Stromal Cells, NGF: Nerve growth factor; NT-3: Neurotrophin-3; PEDF: Pigment epithelium-derived factor; PGIS: Prostaglandin-I synthase; PI3K-C2α: Phosphatidylinositol 3kinase-class II αIsoform; SDF-1: stromal cell-derived factor 1; STAT3: Signal transducer and activator of transcription 3; TGF-β2: Transforming growth factor-beta 2; TIMP-3: tissue inhibitors of metalloproteinases-3; TNFR: tumor necrosis factor receptor; VEGF: Vascular endothelial growth factor.

**RELATED REFERENCES:**

1. Karpov AA, Uspenskaya YK, Minasian SM, Puzanov MV, Dmitrieva RI, Bilibina AA, Anisimov SV, Galagudza MM: **The effect of bone marrow- and adipose tissue-derived mesenchymal stem cell transplantation on myocardial remodelling in the rat model of ischaemic heart failure**. *Int J Exp Pathol* 2013, **94**:169–177.

2. Jiang Z, Hu X, Yu H, Xu Y, Wang L, Chen H, Chen H, Wu R, Zhang Z, Xiang C, Webster KA, Wang J-A: **Human endometrial stem cells confer enhanced myocardial salvage and regeneration by paracrine mechanisms**. *J Cell Mol Med* 2013.

3. Wang Y, Xue M, Xuan Y-L, Hu H-S, Cheng W-J, Suo F, Li X-R, Yan S-H, Wang L-X: **Mesenchymal Stem Cell Therapy Improves Diabetic Cardiac Autonomic Neuropathy and Decreases the Inducibility of Ventricular Arrhythmias**. *Heart Lung Circ* 2013.

4. Bai X, Yan Y, Song Y-H, Seidensticker M, Rabinovich B, Metzele R, Bankson JA, Vykoukal D, Alt E: **Both cultured and freshly isolated adipose tissue-derived stem cells enhance cardiac function after acute myocardial infarction**. *Eur Heart J* 2010, **31**:489–501.

5. Lai RC, Arslan F, Lee MM, Sze NSK, Choo A, Chen TS, Salto-Tellez M, Timmers L, Lee CN, El Oakley RM, Pasterkamp G, de Kleijn DPV, Lim SK: **Exosome secreted by MSC reduces myocardial ischemia/reperfusion injury**. *Stem Cell Res* 2010, **4**:214–222.

6. Hatzistergos KE, Quevedo H, Oskouei BN, Hu Q, Feigenbaum GS, Margitich IS, Mazhari R, Boyle AJ, Zambrano JP, Rodriguez JE, Dulce R, Pattany PM, Valdes D, Revilla C, Heldman AW, McNiece I, Hare JM: **Bone Marrow Mesenchymal Stem Cells Stimulate Cardiac Stem Cell Proliferation and Differentiation**. *Circ Res* 2010, **107**:913–922.

7. Cai L, Johnstone BH, Cook TG, Tan J, Fishbein MC, Chen P-S, March KL: **IFATS Collection: Human Adipose Tissue-Derived Stem Cells Induce Angiogenesis and Nerve Sprouting Following Myocardial Infarction, in Conjunction with Potent Preservation of Cardiac Function**. *STEM CELLS* 2009, **27**:230–237.

8. Schenke-Layland K, Strem BM, Jordan MC, DeEmedio MT, Hedrick MH, Roos KP, Fraser JK, MacLellan WR: **Adipose Tissue-Derived Cells Improve Cardiac Function Following Myocardial Infarction**. *J Surg Res* 2009, **153**:217–223.

9. Li B, Zeng Q, Wang H, Shao S, Mao X, Zhang F, Li S, Guo Z: **Adipose tissue stromal cells transplantation in rats of acute myocardial infarction**. *Coron Artery Dis* 2007, **18**:221–227.

10. Timmers L, Lim SK, Arslan F, Armstrong JS, Hoefer IE, Doevendans PA, Piek JJ, El Oakley RM, Choo A, Lee CN, Pasterkamp G, de Kleijn DPV: **Reduction of myocardial infarct size by human mesenchymal stem cell conditioned medium**. *Stem Cell Res* 2007, **1**:129–137.

11. Uemura R, Xu M, Ahmad N, Ashraf M: **Bone Marrow Stem Cells Prevent Left Ventricular Remodeling of Ischemic Heart Through Paracrine Signaling**. *Circ Res* 2006, **98**:1414–1421.

12. Miyahara Y, Nagaya N, Kataoka M, Yanagawa B, Tanaka K, Hao H, Ishino K, Ishida H, Shimizu T, Kangawa K, Sano S, Okano T, Kitamura S, Mori H: **Monolayered mesenchymal stem cells repair scarred myocardium after myocardial infarction**. *Nat Med* 2006, **12**:459–465.

13. Silva GV, Litovsky S, Assad JAR, Sousa ALS, Martin BJ, Vela D, Coulter SC, Lin J, Ober J, Vaughn WK, Branco RVC, Oliveira EM, He R, Geng Y-J, Willerson JT, Perin EC: **Mesenchymal stem cells differentiate into an endothelial phenotype, enhance vascular density, and improve heart function in a canine chronic ischemia model**. *Circulation* 2005, **111**:150–156.

14. Nagaya N, Fujii T, Iwase T, Ohgushi H, Itoh T, Uematsu M, Yamagishi M, Mori H, Kangawa K, Kitamura S: **Intravenous administration of mesenchymal stem cells improves cardiac function in rats with acute myocardial infarction through angiogenesis and myogenesis**. *Am J Physiol - Heart Circ Physiol* 2004, **287**:H2670–H2676.

15. Liang H, Hou H, Yi W, Yang G, Gu C, Lau WB, Gao E, Ma X, Lu Z, Wei X, Pei J, Yi D: **Increased expression of pigment epithelium-derived factor in aged mesenchymal stem cells impairs their therapeutic efficacy for attenuating myocardial infarction injury**. *Eur Heart J* 2013, **34**:1681–1690.

16. Lu F, Zhao X, Wu J, Cui Y, Mao Y, Chen K, Yuan Y, Gong D, Xu Z, Huang S: **MSCs transfected with hepatocyte growth factor or vascular endothelial growth factor improve cardiac function in the infarcted porcine heart by increasing angiogenesis and reducing fibrosis**. *Int J Cardiol* 2013, **167**:2524–2532.

17. Kearns-Jonker M, Dai W, Gunthart M, Fuentes T, Yeh H-Y, Gerczuk P, Pera M, Mummery C, Kloner RA: **Genetically Engineered Mesenchymal Stem Cells Influence Gene Expression in Donor Cardiomyocytes and the Recipient Heart**. *J Stem Cell Res Ther* 2012, **S1**.

18. Yao J, Jiang S-L, Liu W, Liu C, Chen W, Sun L, Liu K-Y, Jia Z-B, Li R-K, Tian H: **Tissue inhibitor of matrix metalloproteinase-3 or vascular endothelial growth factor transfection of aged human mesenchymal stem cells enhances cell therapy after myocardial infarction**. *Rejuvenation Res* 2012, **15**:495–506.

19. Kim HW, Jiang S, Ashraf M, Haider KH: **Stem cell-based delivery of Hypoxamir-210 to the infarcted heart: implications on stem cell survival and preservation of infarcted heart function**. *J Mol Med Berl Ger* 2012, **90**:997–1010.

20. Cho Y-H, Cha M-J, Song B-W, Kim I-K, Song H, Chang W, Lim S, Ham O, Lee S-Y, Choi E, Kwon HM, Hwang K-C: **Enhancement of MSC adhesion and therapeutic efficiency in ischemic heart using lentivirus delivery with periostin**. *Biomaterials* 2012, **33**:1376–1385.

21. Kim SH, Moon H-H, Kim HA, Hwang K-C, Lee M, Choi D: **Hypoxia-inducible vascular endothelial growth factor-engineered mesenchymal stem cells prevent myocardial ischemic injury**. *Mol Ther J Am Soc Gene Ther* 2011, **19**:741–750.

22. Gao X-R, Tan Y-Z, Wang H-J: **Overexpression of Csx/Nkx2.5 and GATA-4 enhances the efficacy of mesenchymal stem cell transplantation after myocardial infarction**. *Circ J Off J Jpn Circ Soc* 2011, **75**:2683–2691.

23. Lian W-S, Cheng WT-K, Cheng C-C, Hsiao FS-H, Chen J-J, Cheng C-F, Wu S-C: **In vivo therapy of myocardial infarction with mesenchymal stem cells modified with prostaglandin I synthase gene improves cardiac performance in mice**. *Life Sci* 2011, **88**:455–464.

24. Takemiya K, Kai H, Yasukawa H, Tahara N, Kato S, Imaizumi T: **Mesenchymal stem cell-based prostacyclin synthase gene therapy for pulmonary hypertension rats**. *Basic Res Cardiol* 2010, **105**:409–417.

25. Yu Y-S, Shen Z-Y, Ye W-X, Huang H-Y, Hua F, Chen Y-H, Chen K, Lao W-J, Tao L: **AKT-modified autologous intracoronary mesenchymal stem cells prevent remodeling and repair in swine infarcted myocardium**. *Chin Med J (Engl)* 2010, **123**:1702–1708.

26. Huang W, Zhang D, Millard RW, Wang T, Zhao T, Fan G-C, Ashraf A, Xu M, Ashraf M, Wang Y: **Gene manipulated peritoneal cell patch repairs infarcted myocardium**. *J Mol Cell Cardiol* 2010, **48**:702–712.

27. Huang J, Zhang Z, Guo J, Ni A, Deb A, Zhang L, Mirotsou M, Pratt RE, Dzau VJ: **Genetic modification of mesenchymal stem cells overexpressing CCR1 increases cell viability, migration, engraftment, and capillary density in the injured myocardium**. *Circ Res* 2010, **106**:1753–1762.

28. Tsubokawa T, Yagi K, Nakanishi C, Zuka M, Nohara A, Ino H, Fujino N, Konno T, Kawashiri M, Ishibashi-Ueda H, Nagaya N, Yamagishi M: **Impact of anti-apoptotic and anti-oxidative effects of bone marrow mesenchymal stem cells with transient overexpression of heme oxygenase-1 on myocardial ischemia**. *Am J Physiol Heart Circ Physiol* 2010, **298**:H1320–1329.

29. Tang J, Wang J, Zheng F, Kong X, Guo L, Yang J, Zhang L, Huang Y: **Combination of chemokine and angiogenic factor genes and mesenchymal stem cells could enhance angiogenesis and improve cardiac function after acute myocardial infarction in rats**. *Mol Cell Biochem* 2010, **339**:107–118.

30. Eun LY, Song B-W, Cha M-J, Song H, Kim I-K, Choi E, Chang W, Lim S, Choi EJ, Ham O, Lee S-Y, Byun KH, Jang Y, Hwang K-C: **Overexpression of phosphoinositide-3-kinase class II alpha enhances mesenchymal stem cell survival in infarcted myocardium**. *Biochem Biophys Res Commun* 2010, **402**:272–279.

31. Bao C, Guo J, Zheng M, Chen Y, Lin G, Hu M: **Enhancement of the survival of engrafted mesenchymal stem cells in the ischemic heart by TNFR gene transfection**. *Biochem Cell Biol Biochim Biol Cell* 2010, **88**:629–634.

32. Pons J, Huang Y, Takagawa J, Arakawa-Hoyt J, Ye J, Grossman W, Kan YW, Su H: **Combining angiogenic gene and stem cell therapies for myocardial infarction**. *J Gene Med* 2009, **11**:743–753.

33. Zisa D, Shabbir A, Suzuki G, Lee T: **Vascular endothelial growth factor (VEGF) as a key therapeutic trophic factor in bone marrow mesenchymal stem cell-mediated cardiac repair**. *Biochem Biophys Res Commun* 2009, **390**:834–838.

34. Chen SL, Zhu CC, Liu YQ, Tang LJ, Yi L, Yu BJ, Wang DJ: **Mesenchymal stem cells genetically modified with the angiopoietin-1 gene enhanced arteriogenesis in a porcine model of chronic myocardial ischaemia**. *J Int Med Res* 2009, **37**:68–78.

35. Song S-W, Chang W, Song B-W, Song H, Lim S, Kim H-J, Cha M-J, Choi E, Im S-H, Chang B-C, Chung N, Jang Y, Hwang K-C: **Integrin-linked kinase is required in hypoxic mesenchymal stem cells for strengthening cell adhesion to ischemic myocardium**. *Stem Cells Dayt Ohio* 2009, **27**:1358–1365.

36. Deuse T, Peter C, Fedak PWM, Doyle T, Reichenspurner H, Zimmermann WH, Eschenhagen T, Stein W, Wu JC, Robbins RC, Schrepfer S: **Hepatocyte growth factor or vascular endothelial growth factor gene transfer maximizes mesenchymal stem cell-based myocardial salvage after acute myocardial infarction**. *Circulation* 2009, **120**(11 Suppl):S247–254.

37. Gnecchi M, He H, Melo LG, Noiseaux N, Morello F, de Boer RA, Zhang L, Pratt RE, Dzau VJ, Ingwall JS: **Early beneficial effects of bone marrow-derived mesenchymal stem cells overexpressing Akt on cardiac metabolism after myocardial infarction**. *Stem Cells Dayt Ohio* 2009, **27**:971–979.

38. Fan L, Lin C, Zhuo S, Chen L, Liu N, Luo Y, Fang J, Huang Z, Lin Y, Chen J: **Transplantation with survivin-engineered mesenchymal stem cells results in better prognosis in a rat model of myocardial infarction**. *Eur J Heart Fail* 2009, **11**:1023–1030.

39. Wang X, Zhao T, Huang W, Wang T, Qian J, Xu M, Kranias EG, Wang Y, Fan G-C: **Hsp20-engineered mesenchymal stem cells are resistant to oxidative stress via enhanced activation of Akt and increased secretion of growth factors**. *Stem Cells Dayt Ohio* 2009, **27**:3021–3031.

40. Wang M, Tan J, Wang Y, Meldrum KK, Dinarello CA, Meldrum DR: **IL-18 binding protein-expressing mesenchymal stem cells improve myocardial protection after ischemia or infarction**. *Proc Natl Acad Sci U S A* 2009, **106**:17499–17504.
