# Supplementary material for: Mesenchymal stem/stromal cells as a delivery platform in cell and gene therapies
Source: BMC Med. 2015 Aug 12;13:186. doi: 10.1186/s12916-015-0426-0 (PMC4534031; doi:10.1186/s12916-015-0426-0)
Supplement: Additional file 3: — Link 3.1 Overview of the main pre-clinical findings on the impact of wild-type MSC in lung diseases. Link 3.2 Overview of the main pre-clinical findings on the impact of gene-modified MSC in lung diseases. (DOCX 24 kb) [file 12916_2015_426_MOESM3_ESM.docx]

**Link 3.1 Overview of the main pre-clinical findings on the impact of wild type MSC in lung diseases**

| **DISEASE (MODEL)** | **MSC SOURCE** | **TYPE OF STUDY** | **ROUTE OF ADMINISTRATION** | **PROPOSED MECHANISM** | **REF** |
| --- | --- | --- | --- | --- | --- |
| Bronchopulmonary dysplasia  (hyperoxia-induced) | BM | In vitro  In vivo (mouse) | Superficial temporal vein | Paracrine stimulation of endogenous lung stem cells to differentiate and participate in the repair of alveolar injury | [1] |
| Bronchopulmonary dysplasia  (hyperoxia-induced) | Murine BM | In vivo (mouse) | Superficial temporal vein or jugular vein | Treatment with conditioned media ameliorated the pathology | [2] |
| Lung injury  (bleomycin-induced) | Human UC | In vivo (mouse) | Tail vein | Increase in expression of MMP and inhibition of TIMP. Inhibition of the expression of inflammatory cytokines | [3] |
| Fibrotic lung injury  (bleomycin-induced) | Murine BM | In vivo (mouse) | Tail vein | Secretion of humoral factors and cytokines | [4] |
| Bronchopulmonary dysplasia  (hyperoxia-induced) | Murine BM | In vivo (mouse) | Superficial temporal vein | Paracrine release of immunomodulatory factors | [5] |
| Bronchopulmonary dysplasia  (hyperoxia-induced) | Rat BM | In vivo (rat) | Intratracheal | Cell replacing together with paracrine-mediated mechanism | [6] |
| Acute lung injury  (endotoxin-induced) | Murine BM | In vivo (mouse) | Intrapulmonar | Down-regulation of proinflammatory responses (reducing TNF-α and MIP-2 while increasing the anti-inflammatory cytokine IL-10) | [7] |

**Link 3.2 Overview of the main pre-clinical findings on the impact of gene modified MSC in lung diseases**

| **DISEASE (MODEL)** | **MSC SOURCE** | **VECTOR** | **GENE** | **TYPE OF STUDY** | **ROUTE OF ADMINISTRATION** | **PROPOSED MECHANISM** | **REF** |
| --- | --- | --- | --- | --- | --- | --- | --- |
| Acute lung injury, Acute respiratory distress syndrome  (lipopolysaccharide-induced) | Murine BM | Non-viral | Human Ang-1 | In vivo (mouse) | Jugular vein | Reduced pulmonary inflammation, reduced proinflammatory cytokines, increased secretion, increased lung permeability, immunomodulatory function, reduced endothelial cell activation | [8] |
| Acute lung injury (lipopolysaccharide-induced) | Murine BM | Lentiviral | Ang-1 | In vivo (mouse) | Jugular vein | Increased secretion, injury improved, attenuate inflammatory reaction and vascular leakage, reduced pulmonary permeability and down-regulation of pro-inflammatory genes | [9] |
| Lung injury  (ischemia-reperfusion-induced) | Rat BM | Retroviral | IL-10 | In vivo (rat) | Penile vein | Lung microvascular permeability reduced, reduced apoptosis, functional improvement | [10] |
| Pulmonary hypertension  (monocrotaline-induced) | Rat BM | Retroviral | Prostacyclin synthase | In vivo (rat) | Tail vein | Increased mice survival, functional improvement | [11] |
| RILI (Radiation-induced) | Murine BM | Adenoviral | TGF-β | In vivo (mouse) | Tail vein | Migration, protection, alleviated lung injury, paracrine mechanisms, homing, modulate inflammatory responses | [12] |

**Abbreviations:** Ang-1: Angiopoietin 1; BM: Bone marrow; IL-10: Interleukin-10; MIP-2: macrophage inflammatory protein-2; MMP: Matrix metalloproteinases; TIMP: Tissue inhibitors of metalloproteinases; TNF-α: Tumor necrosis factor-alpha; UC: Umbilical cord; RILI: Radiation induced lung injury.

**RELATED REFERENCES:**

1. Tropea KA, Leder E, Aslam M, Lau AN, Raiser DM, Lee J-H, Balasubramaniam V, Fredenburgh LE, Alex Mitsialis S, Kourembanas S, Kim CF: **Bronchioalveolar stem cells increase after mesenchymal stromal cell treatment in a mouse model of bronchopulmonary dysplasia**. *Am J Physiol Lung Cell Mol Physiol* 2012, **302**:L829–837.

2. Hansmann G, Fernandez-Gonzalez A, Aslam M, Vitali SH, Martin T, Mitsialis SA, Kourembanas S: **Mesenchymal stem cell-mediated reversal of bronchopulmonary dysplasia and associated pulmonary hypertension**. *Pulm Circ* 2012, **2**:170–181.

3. Moodley Y, Atienza D, Manuelpillai U, Samuel CS, Tchongue J, Ilancheran S, Boyd R, Trounson A: **Human Umbilical Cord Mesenchymal Stem Cells Reduce Fibrosis of Bleomycin-Induced Lung Injury**. *Am J Pathol* 2009, **175**:303–313.

4. Kumamoto M, Nishiwaki T, Matsuo N, Kimura H, Matsushima K: **Minimally cultured bone marrow mesenchymal stem cells ameliorate fibrotic lung injury**. *Eur Respir J* 2009, **34**:740–748.

5. Aslam M, Baveja R, Liang OD, Fernandez-Gonzalez A, Lee C, Mitsialis SA, Kourembanas S: **Bone marrow stromal cells attenuate lung injury in a murine model of neonatal chronic lung disease**. *Am J Respir Crit Care Med* 2009, **180**:1122–1130.

6. Van Haaften T, Byrne R, Bonnet S, Rochefort GY, Akabutu J, Bouchentouf M, Rey-Parra GJ, Galipeau J, Haromy A, Eaton F, Chen M, Hashimoto K, Abley D, Korbutt G, Archer SL, Thébaud B: **Airway delivery of mesenchymal stem cells prevents arrested alveolar growth in neonatal lung injury in rats**. *Am J Respir Crit Care Med* 2009, **180**:1131–1142.

7. Gupta N, Su X, Popov B, Lee JW, Serikov V, Matthay MA: **Intrapulmonary Delivery of Bone Marrow-Derived Mesenchymal Stem Cells Improves Survival and Attenuates Endotoxin-Induced Acute Lung Injury in Mice**. *J Immunol* 2007, **179**:1855–1863.

8. Mei SHJ, McCarter SD, Deng Y, Parker CH, Liles WC, Stewart DJ: **Prevention of LPS-induced acute lung injury in mice by mesenchymal stem cells overexpressing angiopoietin 1**. *PLoS Med* 2007, **4**:e269.

9. Xu J, Qu J, Cao L, Sai Y, Chen C, He L, Yu L: **Mesenchymal stem cell-based angiopoietin-1 gene therapy for acute lung injury induced by lipopolysaccharide in mice**. *J Pathol* 2008, **214**:472–481.

10. Manning E, Pham S, Li S, Vazquez-Padron RI, Mathew J, Ruiz P, Salgar SK: **Interleukin-10 delivery via mesenchymal stem cells: a novel gene therapy approach to prevent lung ischemia-reperfusion injury**. *Hum Gene Ther* 2010, **21**:713–727.

11. Takemiya K, Kai H, Yasukawa H, Tahara N, Kato S, Imaizumi T: **Mesenchymal stem cell-based prostacyclin synthase gene therapy for pulmonary hypertension rats**. *Basic Res Cardiol* 2010, **105**:409–417.

12. Xue J, Li X, Lu Y, Gan L, Zhou L, Wang Y, Lan J, Liu S, Sun L, Jia L, Mo X, Li J: **Gene-modified mesenchymal stem cells protect against radiation-induced lung injury**. *Mol Ther J Am Soc Gene Ther* 2013, **21**:456–465.
