# Supplementary material for: Mesenchymal stem/stromal cells as a delivery platform in cell and gene therapies
Source: BMC Med. 2015 Aug 12;13:186. doi: 10.1186/s12916-015-0426-0 (PMC4534031; doi:10.1186/s12916-015-0426-0)
Supplement: Additional file 4: — Link 4.1 Overview of the main pre-clinical findings on the impact of wild-type MSC in liver diseases. Link 4.2 Overview of the main pre-clinical findings on the impact of gene-modified MSC in liver diseases. (DOCX 22 kb) [file 12916_2015_426_MOESM4_ESM.docx]

**Link 4.1 Overview of the main pre-clinical findings on the impact of wild type MSC in liver diseases**

| **DISEASE (MODEL)** | **MSC SOURCE** | **TYPE OF STUDY** | **ROUTE OF ADMINISTRATION** | **PROPOSED MECHANISM** | **REF** |
| --- | --- | --- | --- | --- | --- |
| Liver fibrosis  (CCl_4_ induced) | Murine  BM | In vivo (mouse) | Tail vein | Paracrine and immunomodulatory effect (HGF, VEGF, NGF) | [1] |
| Liver fibrosis  (CCl_4_ induced) | Murine BM | In vivo (mouse) | Tail vein | Antioxidative effect by increasing SOD activity and inhibiting ROS production. Increased gene expression levels of Hmox-1, BI-1, HGF, GST and Nrf2 | [2] |
| Liver fibrosis  (CCl_4_ induced) | Murine  BM | In vivo (mouse) | Tail vein | Dlk1 expression inhibition | [3] |
| Ischemia reperfusion injury (hepatectomy plus ischemia-reperfusion) | Rat  BM | In vivo  (rat) | Portal vein | Putative paracrine action | [4] |
| Liver fibrosis  (CCl_4_ induced) | Human  UC | In vivo  (rat) | Intrahepatic | Secretion of various bioactive cytokines (in particular T cell-attracting chemokine, leukemia inhibitory factor, and prolactin) | [5] |
| Liver fibrosis  (CCl_4_ induced) | Human  UC | In vivo (mouse) | Intrahepatic | Differentiation into hepatocytes and HGF production | [6] |
| Acute liver failure  (CCl_4_ induced) | Human  AD | In vivo (mouse) | Tail vein | Secretion of bioactive factors (immunosuppressive, hepatocyte-growth promoting, hematopoiesis promoting) | [7] |
| Fulminant hepatic failure (hepatotoxin induced) | Human  BM | In vivo  (rat) | Penile vein or extracorporeal perfusion | Various paracrine mediators, including immunomodulatory ones (IGFBP-1, leptin, CCL2, etc) | [8] |

**Link 4.2 Overview of the main pre-clinical findings on the impact of gene modified MSC in liver diseases**

| **DISEASE (MODEL)** | **MSC SOURCE** | **VECTOR** | **GENE** | **TYPE OF STUDY** | **ROUTE OF ADMINISTRATION** | **PROPOSED MECHANISM** | **REF** |
| --- | --- | --- | --- | --- | --- | --- | --- |
| Liver transplantation  (50% reduced size liver transplantation) | Rat BM | Adenoviral | CXCR4 | In vivo  (rat) | Penile vein | Increased engraftment, improved proliferation, increased secretion/release of cytokines by paracrine mechanisms, migration, enhanced mobilization and homing, early regeneration | [9] |
| Liver disease  (Monocrotaline-induced and 70% reduced liver) | Murine AD | AAV | Human AAT1 | In vivo (mouse) | Intrasplenic | Regeneration, long-term transgene expression, homing, avoided host immune response | [10] |
| Liver fibrosis | N/A | Plasmid | HGF | In vivo  (rat) | Tail vein | HGF accumulation in liver, decreased fibrosis, secretion, migration | [11] |

**Abbreviations:** AAT1:Aspartate aminotransferase-1; AAV: Adeno-associated virus; AD: Adipose; BI-1: Bax inhibitor-1; BM: Bone marrow; CCL2: chemokine (C-C motif) ligand 2; CXCR4: C-X-C chemokine receptor type; Dlk1: Delta-like 1 homolog; GST: glutathione S-transferase; IGFBP-1: Insulin-like growth factor-binding protein 1; Nrf2: Nuclear factor (erythroid-derived 2)-like 2; HGF: Hepatocyte growth factor; Hmox-1: heme oxygenase-1; NGF: Nerve growth factor ; ROS: Reactive oxygen species; SOD: Superoxide dismutase; UC: Umbilical cord; VEGF: Vascular endothelial growth factor.

**RELATED REFERENCES**

1. Li Q, Zhou X, Shi Y, Li J, Zheng L, Cui L, Zhang J, Wang L, Han Z, Han Y, Fan D: **In vivo tracking and comparison of the therapeutic effects of MSCs and HSCs for liver injury**. *PloS One* 2013, **8**:e62363.

2. Cho K, Woo S, Seoh J, Han H, Ryu K: **Mesenchymal stem cells restore CCl _4_ -induced liver injury by an antioxidative process**. *Cell Biol Int* 2012, **36**:1267–1274.

3. Pan R-L, Wang P, Xiang L-X, Shao J-Z: **Delta-like 1 Serves as a New Target and Contributor to Liver Fibrosis Down-regulated by Mesenchymal Stem Cell Transplantation**. *J Biol Chem* 2011, **286**:12340–12348.

4. Kanazawa H, Fujimoto Y, Teratani T, Iwasaki J, Kasahara N, Negishi K, Tsuruyama T, Uemoto S, Kobayashi E: **Bone Marrow-Derived Mesenchymal Stem Cells Ameliorate Hepatic Ischemia Reperfusion Injury in a Rat Model**. *PLoS ONE* 2011, **6**:e19195.

5. Tsai P-C, Fu T-W, Chen Y-MA, Ko T-L, Chen T-H, Shih Y-H, Hung S-C, Fu Y-S: **The therapeutic potential of human umbilical mesenchymal stem cells from Wharton’s jelly in the treatment of rat liver fibrosis**. *Liver Transplant Off Publ Am Assoc Study Liver Dis Int Liver Transplant Soc* 2009, **15**:484–495.

6. Yan Y, Xu W, Qian H, Si Y, Zhu W, Cao H, Zhou H, Mao F: **Mesenchymal stem cells from human umbilical cords ameliorate mouse hepatic injury in vivo**. *Liver Int* 2009, **29**:356–365.

7. Banas A, Teratani T, Yamamoto Y, Tokuhara M, Takeshita F, Osaki M, Kawamata M, Kato T, Okochi H, Ochiya T: **IFATS Collection: In Vivo Therapeutic Potential of Human Adipose Tissue Mesenchymal Stem Cells After Transplantation into Mice with Liver Injury**. *STEM CELLS* 2008, **26**:2705–2712.

8. Parekkadan B, van Poll D, Suganuma K, Carter EA, Berthiaume F, Tilles AW, Yarmush ML: **Mesenchymal stem cell-derived molecules reverse fulminant hepatic failure**. *PloS One* 2007, **2**:e941.

9. Du Z, Wei C, Yan J, Han B, Zhang M, Peng C, Liu Y: **Mesenchymal stem cells overexpressing C-X-C chemokine receptor type 4 improve early liver regeneration of small-for-size liver grafts**. *Liver Transplant Off Publ Am Assoc Study Liver Dis Int Liver Transplant Soc* 2013, **19**:215–225.

10. Li H, Zhang B, Lu Y, Jorgensen M, Petersen B, Song S: **Adipose tissue-derived mesenchymal stem cell-based liver gene delivery**. *J Hepatol* 2011, **54**:930–938.

11. Ishikawa H, Jo J-I, Tabata Y: **Liver Anti-Fibrosis Therapy with Mesenchymal Stem Cells Secreting Hepatocyte Growth Factor**. *J Biomater Sci Polym Ed* 2012, **23**:2259–2272.
