# Supplementary material for: Mesenchymal stem/stromal cells as a delivery platform in cell and gene therapies
Source: BMC Med. 2015 Aug 12;13:186. doi: 10.1186/s12916-015-0426-0 (PMC4534031; doi:10.1186/s12916-015-0426-0)
Supplement: Additional file 5: — Link 5.1 Overview of the main pre-clinical findings on the impact of wild-type MSC in pancreatic diseases. Link 5.2 Overview of the main pre-clinical findings on the impact of gene-modified MSC in pancreatic diseases. (DOCX 20 kb) [file 12916_2015_426_MOESM5_ESM.docx]

**Link 5.1 Overview of the main pre-clinical findings on the impact of wild type MSC in pancreatic diseases**

| **DISEASE & MODEL** | **MSC SOURCE** | **TYPE OF STUDY** | **ROUTE OF ADMINISTRATION** | **PROPOSED MECHANISM** | **REF** |
| --- | --- | --- | --- | --- | --- |
| Type 1 diabetes mellitus  (STZ-induced) | Murine  BM | In vivo (mouse) | Tail vein | Restore the systemic and local balance between autoaggressive and regulatory T cells, cause a shift from proinflammatory to antinflammatory cytokines | [1] |
| Type 1 diabetes mellitus  (STZ-induced) | Murine  BM | In vivo (mouse)  In vitro | Tail vein | MSC-conditioned medium exerts a striking protective effect on in vivo diabetic islets and on isolated islets exposed to STZ in vitro | [2] |
| Type 2 diabetes mellitus  (fat-fed/STZ-induced) | Human BM | In vivo (rat) | Tail vein | Improve insulin sensitivity and restore the concentration of GLUT4  glucose transporter | [3] |
| Pancreatitis  (cerulein-induced Mild AP and sodium taurocholate solution-induced Severe AP) | Human BM | In vivo (rat) | Tail vein | Recover pancreas function by decreasing the expression of inflammatory mediators/cytokines and inhibiting T-cell infiltration, as well as up-regulating expression of regulatory T cells | [4] |

**Link 5.2 Overview of the main pre-clinical findings on the impact of gene modified MSC in pancreatic diseases**

| **DISEASE & MODEL** | **MSC SOURCE** | **VECTOR** | **GENE** | **TYPE OF STUDY** | **ROUTE OF ADMINISTRATION** | **PROPOSED MECHANISM** | **REF** |
| --- | --- | --- | --- | --- | --- | --- | --- |
| Type 1 diabetes mellitus  (STZ-induced) | Human BM | Plasmid | Human Insulin under human EGR1 promoter | In vivo  (mouse) | Intrahepatic or intraperitoneal transplantation | Safe and efficient insulin-secreting bioimplants for diabetes treatment | [5] |
| Type 1 diabetes mellitus  (STZ-induced) | Human BM | Adenoviral | Human HGF and human IL-1Ra | In vivo  (mouse) | Transplantation under the kidney capsule | Promote local revascularization, improve viability of co-transplanted islets and reversed diabetes in long term | [6] |
| Type 1 diabetes mellitus  (STZ-induced) | Human BM | Adenoviral | Human VEGF and human IL-1Ra | In vivo  (mouse) | Transplantation under the kidney capsule | Protect islet viability, promote revascularization and  significantly improve the glycemic control | [7] |

**Abbreviations:** AP: Acute pancreatitis; BM: Bone marrow; EGR1: Early growth response protein 1; GLUT4: Glucose transporter4; HGF: Hepatocyte growth factor; IL-1Ra: Interleukin-1 receptor antagonist; STZ: Streptozotocin.

**RELATED REFERENCES**

1. Ezquer F, Ezquer M, Contador D, Ricca M, Simon V, Conget P: **The Antidiabetic Effect of Mesenchymal Stem Cells Is Unrelated to Their Transdifferentiation Potential But to Their Capability to Restore Th1/Th2 Balance and to Modify the Pancreatic Microenvironment**. *STEM CELLS* 2012, **30**:1664–1674.

2. Gao X, Song L, Shen K, Wang H, Qian M, Niu W, Qin X: **Bone marrow mesenchymal stem cells promote the repair of islets from diabetic mice through paracrine actions**. *Mol Cell Endocrinol* 2014, **388**:41–50.

3. Si Y, Zhao Y, Hao H, Liu J, Guo Y, Mu Y, Shen J, Cheng Y, Fu X, Han W: **Infusion of Mesenchymal Stem Cells Ameliorates Hyperglycemia in Type 2 Diabetic Rats: Identification of a Novel Role in Improving Insulin Sensitivity**. *Diabetes* 2012, **61**:1616–1625.

4. Jung KH, Song SU, Yi T, Jeon M, Hong S, Zheng H, Lee H, Choi M, Lee D, Hong S: **Human Bone Marrow–Derived Clonal Mesenchymal Stem Cells Inhibit Inflammation and Reduce Acute Pancreatitis in Rats**. *Gastroenterology* 2011, **140**:998–1008.e4.

5. Chen NKF, Tan SY, Udolph G, Kon OL: **Insulin expressed from endogenously active glucose-responsive EGR1 promoter in bone marrow mesenchymal stromal cells as diabetes therapy**. *Gene Ther* 2010, **17**:592–605.

6. Wu H, Lu W, Mahato RI: **Mesenchymal stem cells as a gene delivery vehicle for successful islet transplantation**. *Pharm Res* 2011, **28**:2098–2109.

7. Mundra V, Wu H, Mahato RI: **Genetically Modified Human Bone Marrow Derived Mesenchymal Stem Cells for Improving the Outcome of Human Islet Transplantation**. *PLoS ONE* 2013, **8**:e77591.
