# Supplementary material for: Mesenchymal stem/stromal cells as a delivery platform in cell and gene therapies
Source: BMC Med. 2015 Aug 12;13:186. doi: 10.1186/s12916-015-0426-0 (PMC4534031; doi:10.1186/s12916-015-0426-0)
Supplement: Additional file 6: — Link 6.1 Overview of the main pre-clinical findings on the impact of wild-type MSC in renal diseases. Link 6.2 Overview of the main pre-clinical findings on the impact of gene-modified MSC in renal diseases. (DOCX 29 kb) [file 12916_2015_426_MOESM6_ESM.docx]

**Link 6.1 Overview of the main pre-clinical findings on the impact of wild type MSC in renal diseases**

| **DISEASE (MODEL)** | **MSC SOURCE** | **TYPE OF STUDY** | **ROUTE OF ADMINISTRATION** | **PROPOSED MECHANISM** | **REF** |
| --- | --- | --- | --- | --- | --- |
| Acute kidney injury  (ischemia reperfusion-induced) | Murine BM | In vivo (mouse) | Tail vein | Reduction in loss of peritubular capillaries and tubular injury, promotion of parenchymal cell proliferation, decrease in macrophage infiltration and decrease of apoptotic cells | [1] |
| Acute kidney injury  (ischemia reperfusion-induced) | Human WJ | In vivo (rat) | Tail vein | Proliferation, apoptosis reduced, decreased macrophages, renal fibrosis inhibited, improved renal function | [2] |
| Acute kidney injury (cisplatin-induced) | Human UC | In vivo (rat) | Injection into renal capsule | Serum creatinine, blood urea nitrogen levels decreased, increased proliferation, repaired cell injury | [3] |
| Diabetic nephropathy  (streptozotocin-induced) | Human AD | In vivo (rat) | Tail vein | Secretion of TSG-6, FGF2, EGF, GDNF | [4] |
| Acute kidney injury (cisplatin-induced) | Human BM | In vivo (mouse) | Tail vein | Enhanced survival, ameliorated renal function, upregulation of anti-apoptotic genes and down-regulation of apoptotic genes | [5] |
| Acute kidney injury (gentamicin-induced) | Rat BM | In vivo (rat) | Intravenous | Functional repair | [6] |
| Kidney injury (nephrectomy) | Murine BM | In vivo (mouse) | Tail vein | Serum creatinine, uric acid, proteinurea levels decreased, less fibrosis and tubular atrophy | [7] |
| Acute kidney injury  (ischemia reperfusion-induced) | Human BM | In vivo (rat) | Intravenous | Protection, reduced apoptosis, promoted survival, reduced impairment of renal function | [8] |
| Acute kidney injury  (cisplatin-induced) | Human UCB | In vivo (mouse) | Intravenous | Expression of growth factors with mitogenic and anti-inflammatory action (especially HGF) and other molecules that in turn stimulate target cells to produce growth factors with regenerative potential for renal cells | [9] |
| Acute kidney injury (glycerol-induced) | Human BM | In vivo (mouse) | Tail vein | Amelioration of renal function, reduced fibrosis, proliferation, reduced apoptosis, functional recovery | [10] |
| Diabetic nephropathy  (streptozotocin-induced) | Murine BM | In vivo (mouse) | Tail vein | Increase in the number of insulin-producing cells and restriction of glucagon-producing cell expansion | [11] |
| Glomerular pathology (ColA2 deficiency) | Human fetal peripheral blood | In vivo (mouse) | Intrauterine | Supply of collagen lacking chain to host cells | [12] |
| Renal injury  (cisplatin-induced) | Human BM | In vivo (mouse) | Tail vein | Secretion of pro-survival growth factors (IGF-1) and anti-inflammatory effect | [13] |
| Acute kidney injury  (cisplatin-induced) | Murine BM | In vivo (mouse) | Intravenous | Secretion of IGF-1 | [14] |
| Glomerulonephritis  (anti-Thy1.1-induced) | Rat BM | In vivo (rat) | Intra-artery | Secretion of VEGF, TGF-β, HGF | [15] |
| Acute renal failure  (ischemia reperfusion-induced) | Rat BM | In vivo (rat) | N/A | Increase in anti-inflammatory cytokines | [16] |
| Glomerulonephritis  (anti-Thy1.1-induced) | Rat BM | In vivo (rat) | Intra-artery and tail vein | Paracrine effect on neighboring glomerular cells, secretion of chemoattractors and “feeders” to circulating hematopoietic stem cells | [17] |
| Alport syndrome  (Col4A3-deficient mouse) | Murine BM | In vivo (mouse) | Intravenous | Secretion of VEGF and BMP-7 | [18] |

**Link 6.2 Overview of the main pre-clinical findings on the impact of gene modified MSC in renal diseases**

| **DISEASE (MODEL)** | **MSC SOURCE** | **VECTOR** | **GENE** | **TYPE OF STUDY** | **ROUTE OF ADMINISTRATION** | **PROPOSED MECHANISM** | **REF** |
| --- | --- | --- | --- | --- | --- | --- | --- |
| Acute renal failure  (ischemia reperfusion-induced) | Rabbit BM | Adenoviral | Human BMP-7 | In vivo (Rabbit) | Renal artery | Co-operative effect involving survival, mobilization and homing, immune modulatory capacity, functional recovery, paracrine mechanisms, proliferation, inhibit apoptosis, migration, differentiation, regeneration | [19] |
| Glomerulonephritis (nephrotoxic serum-induced) | Human BM | Adenoviral | Human GDNF | In vivo (rat) | Renal artery | Migration, ameliorated renal function | [20] |
| Anemia  (electrocoagulation-induced) | Murine BM | Retroviral | Mouse EPO; mouse IGF-1A | In vivo (mouse) | Subcutaneous | Paracrine support, secretion, improved MSC survival, increased hematocrit, reduced apoptosis, functional recovery | [21] |
| Acute renal failure  (ischemia-reperfusion-induced) | Rat BM | Adenoviral | Human Tissue Kallikrein | In vivo (rat) | Carotid artery | Migration, protection, secretion, improved survival, antioxidative, antiapoptotic, antiinflammatory and angiogenic effects, autocrine and paracrine actions | [22] |
| Anemia  (electrocoagulation-induced) | Murine BM | Retroviral | Mouse  EPO | In vivo (mouse) | Subcutaneous | Secretion, increased hematocrit, functional recovery | [23] |

**Abbreviations:** AD: Adipose; BM: Bone marrow; BMP-7: Bone morphogenetic protein 7; EGF: Epidermal growth factor; EPO: Erythropoietin; FGF2: Fibroblast growth factor-2; GDNF: Glial cell-derived neurotrophic factor; HGF: Hepatocyte growth factor; IGF-1: Insulin-like growth factor-1; TGF-β: Transforming growth factor-beta; TSG-6: Tumor necrosis factor-inducible gene-6; UC: Umbilical cord; UCB: Umbilical cord blood; VEGF: Vascular endothelial growth factor; WJ: Wharton’s Jelly.

**RELATED REFERENCES**

1. Xing L, Cui R, Peng L, Ma J, Chen X, Xie R-J, Li B: **Mesenchymal stem cells, not conditioned medium, contribute to kidney repair after ischemia-reperfusion injury**. *Stem Cell Res Ther* 2014, **5**.

2. Zou X, Zhang G, Cheng Z, Yin D, Du T, Ju G, Miao S, Liu G, Lu M, Zhu Y: **Microvesicles derived from human Wharton’s Jelly mesenchymal stromal cells ameliorate renal ischemia-reperfusion injury in rats by suppressing CX3CL1**. *Stem Cell Res Ther* 2014, **5**:40.

3. Zhou Y, Xu H, Xu W, Wang B, Wu H, Tao Y, Zhang B, Wang M, Mao F, Yan Y, Gao S, Gu H, Zhu W, Qian H: **Exosomes released by human umbilical cord mesenchymal stem cells protect against cisplatin-induced renal oxidative stress and apoptosis in vivo and in vitro**. *Stem Cell Res Ther* 2013, **4**:34.

4. Zhang L, Li K, Liu X, Li D, Luo C, Fu B, Cui S, Zhu F, Zhao RC, Chen X: **Repeated Systemic Administration of Human Adipose-Derived Stem Cells Attenuates Overt Diabetic Nephropathy in Rats**. *Stem Cells Dev* 2013:130821130132003.

5. Bruno S, Grange C, Collino F, Deregibus MC, Cantaluppi V, Biancone L, Tetta C, Camussi G: **Microvesicles derived from mesenchymal stem cells enhance survival in a lethal model of acute kidney injury**. *PloS One* 2012, **7**:e33115.

6. Reis LA, Borges FT, Simoes MJ, Borges AA, Sinigaglia-Coimbra R, Schor N: **Bone Marrow-Derived Mesenchymal Stem Cells Repaired but Did Not Prevent Gentamicin-Induced Acute Kidney Injury through Paracrine Effects in Rats**. *PLoS ONE* 2012, **7**.

7. He J, Wang Y, Sun S, Yu M, Wang C, Pei X, Zhu B, Wu J, Zhao W: **Bone marrow stem cells-derived microvesicles protect against renal injury in the mouse remnant kidney model**. *Nephrology* 2012, **17**:493–500.

8. Gatti S, Bruno S, Deregibus MC, Sordi A, Cantaluppi V, Tetta C, Camussi G: **Microvesicles derived from human adult mesenchymal stem cells protect against ischaemia-reperfusion-induced acute and chronic kidney injury**. *Nephrol Dial Transplant Off Publ Eur Dial Transpl Assoc - Eur Ren Assoc* 2011, **26**:1474–1483.

9. Morigi M, Rota C, Montemurro T, Montelatici E, Lo Cicero V, Imberti B, Abbate M, Zoja C, Cassis P, Longaretti L, Rebulla P, Introna M, Capelli C, Benigni A, Remuzzi G, Lazzari L: **Life-Sparing Effect of Human Cord Blood-Mesenchymal Stem Cells in Experimental Acute Kidney Injury**. *STEM CELLS* 2010, **28**:513–522.

10. Bruno S, Grange C, Deregibus MC, Calogero RA, Saviozzi S, Collino F, Morando L, Busca A, Falda M, Bussolati B, Tetta C, Camussi G: **Mesenchymal stem cell-derived microvesicles protect against acute tubular injury**. *J Am Soc Nephrol JASN* 2009, **20**:1053–1067.

11. Ezquer FE, Ezquer ME, Parrau DB, Carpio D, Yañez AJ, Conget PA: **Systemic Administration of Multipotent Mesenchymal Stromal Cells Reverts Hyperglycemia and Prevents Nephropathy in Type 1 Diabetic Mice**. *Biol Blood Marrow Transplant* 2008, **14**:631–640.

12. Guillot P, Cook H, Pusey C, Fisk N, Harten S, Moss J, Shore I, Bou-Gharios G: **Transplantation of human fetal mesenchymal stem cells improves glomerulopathy in a collagen type Iα2-deficient mouse**. *J Pathol* 2008, **214**:627–636.

13. Morigi M, Introna M, Imberti B, Corna D, Abbate M, Rota C, Rottoli D, Benigni A, Perico N, Zoja C, Rambaldi A, Remuzzi A, Remuzzi G: **Human Bone Marrow Mesenchymal Stem Cells Accelerate Recovery of Acute Renal Injury and Prolong Survival in Mice**. *STEM CELLS* 2008, **26**:2075–2082.

14. Imberti B, Morigi M, Tomasoni S, Rota C, Corna D, Longaretti L, Rottoli D, Valsecchi F, Benigni A, Wang J, Abbate M, Zoja C, Remuzzi G: **Insulin-Like Growth Factor-1 Sustains Stem Cell–Mediated Renal Repair**. *J Am Soc Nephrol* 2007, **18**:2921–2928.

15. Kunter U, Rong S, Boor P, Eitner F, Müller-Newen G, Djuric Z, Roeyen CR van, Konieczny A, Ostendorf T, Villa L, Milovanceva-Popovska M, Kerjaschki D, Floege J: **Mesenchymal Stem Cells Prevent Progressive Experimental Renal Failure but Maldifferentiate into Glomerular Adipocytes**. *J Am Soc Nephrol* 2007, **18**:1754–1764.

16. Semedo P, Wang PM, Andreucci TH, Cenedeze MA, Teixeira VPA, Reis MA, Pacheco-Silva A, Câmara NOS: **Mesenchymal Stem Cells Ameliorate Tissue Damages Triggered by Renal Ischemia and Reperfusion Injury**. *Transplant Proc* 2007, **39**:421–423.

17. Kunter U, Rong S, Djuric Z, Boor P, Müller-Newen G, Yu D, Floege J: **Transplanted Mesenchymal Stem Cells Accelerate Glomerular Healing in Experimental Glomerulonephritis**. *J Am Soc Nephrol* 2006, **17**:2202–2212.

18. Ninichuk V, Gross O, Segerer S, Hoffmann R, Radomska E, Buchstaller A, Huss R, Akis N, Schlöndorff D, Anders H-J: **Multipotent mesenchymal stem cells reduce interstitial fibrosis but do not delay progression of chronic kidney disease in collagen4A3-deficient mice**. *Kidney Int* 2006, **70**:121–129.

19. Zhen-Qiang F, Bing-Wei Y, Yong-Liang L, Xiang-Wei W, Shan-Hong Y, Yuan-Ning Z, Wei-Sheng J, Wei C, Ye G: **Localized expression of human BMP-7 by BM-MSCs enhances renal repair in an in vivo model of ischemia-reperfusion injury**. *Genes Cells Devoted Mol Cell Mech* 2012, **17**:53–64.

20. Huang Z-Y, Hong L-Q, Na N, Luo Y, Miao B, Chen J: **Infusion of mesenchymal stem cells overexpressing GDNF ameliorates renal function in nephrotoxic serum nephritis**. *Cell Biochem Funct* 2012, **30**:139–144.

21. Kucic T, Copland IB, Cuerquis J, Coutu DL, Chalifour LE, Gagnon RF, Galipeau J: **Mesenchymal stromal cells genetically engineered to overexpress IGF-I enhance cell-based gene therapy of renal failure-induced anemia**. *Am J Physiol Renal Physiol* 2008, **295**:F488–496.

22. Hagiwara M, Shen B, Chao L, Chao J: **Kallikrein-modified mesenchymal stem cell implantation provides enhanced protection against acute ischemic kidney injury by inhibiting apoptosis and inflammation**. *Hum Gene Ther* 2008, **19**:807–819.

23. Eliopoulos N, Gagnon RF, Francois M, Galipeau J: **Erythropoietin delivery by genetically engineered bone marrow stromal cells for correction of anemia in mice with chronic renal failure**. *J Am Soc Nephrol JASN* 2006, **17**:1576–1584.
