# Supplementary material for: Mesenchymal stem/stromal cells as a delivery platform in cell and gene therapies
Source: BMC Med. 2015 Aug 12;13:186. doi: 10.1186/s12916-015-0426-0 (PMC4534031; doi:10.1186/s12916-015-0426-0)
Supplement: Additional file 7: — Link 7.1 Overview of the main pre-clinical findings on the impact of wild-type MSC in skeletal diseases. Link 7.2 Overview of the main pre-clinical findings on the impact of gene-modified MSC in skeletal diseases. (DOCX 24 kb) [file 12916_2015_426_MOESM7_ESM.docx]

**Link 7.1 Overview of the main pre-clinical findings on the impact of wild type MSC in skeletal diseases**

| **DISEASE (MODEL)** | **MSC SOURCE** | **TYPE OF STUDY** | **ROUTE OF ADMINISTRATION** | **PROPOSED MECHANISM** | **REF** |
| --- | --- | --- | --- | --- | --- |
| Osteogenesis imperfecta | Murine BM  Human BM | In vivo (mouse) and clinical trial | Tail vein | Secretion of soluble mediators that initiate a biochemical pathway involving several intermediaries | [1] |
| Rheumatoid arthritis  (collagen-induced) | Human UCB | In vivo  (rat) | Intra-articular | Downregulation of TNF-α, IL-1 and IFN-γ, upregulation of IL-10 | [2] |
| Rheumatoid arthritis  (collagen-induced) | Rat BM | In vivo (mouse) | Intravenous | Decrease in serum levels of pro-inflammatory cytokine, TNF-α and inflammatory cell infiltration. Increased expression of anti-inflammatory cytokines IL-10, IP-10, and CXCR3 | [3] |
| Rheumatoid arthritis  (collagen-induced) | Murine BM | In vivo (mouse) | Intraperitoneal | MSC induced hyporesponsiveness of T lymphocytes, modulated the expression of inflammatory cytokines (TNF-α) and exerted immunomodulatory function by educating antigen-specific Tregs | [4] |

**Link 7.2 Overview of the main pre-clinical findings on the impact of gene modified MSC in skeletal diseases**

| **DISEASE (MODEL)** | **MSC SOURCE** | **VECTOR** | **GENE** | **TYPE OF STUDY** | **ROUTE OF ADMINISTRATION** | **PROPOSED MECHANISM** | **REF** |
| --- | --- | --- | --- | --- | --- | --- | --- |
| Cartilage repair  (nude mouse) | Human Dental | RGD-coupled alginate microspheres | TGFβ-1 | In vivo (mouse) | Subcutaneous | Secretion, chondrogenic differentiation, cartilage tissue regeneration | [5] |
| Mandible bone distraction (bone distraction) | N/A | Adenoviral | BMP-2 | In vivo (dog) | N/A | Increased mineralization, improved bone remodeling and maturation | [6] |
| Cartilage repair (osteochondral defects induced) | Human BM | Lentiviral | Human ZNF145, siRNA(ZNF145) | In vivo (rat) | Transplanted into knee | Enhanced chondrogenesis, improved repair | [7] |
| Cartilage repair (cartilage defects induced) | Rabbit BM | Adenoviral | SOX-9 | In vivo (rabbit) | Transplanted into knee | Greater expression of chondrogenic marker genes, improved repair | [8] |
| Tendon repair  (rotator cuff) | Rat BM | Adenoviral | Scleraxis | In vivo (rat) | Implanted into rotator cuff repair site | Augmented rotator cuff healing and reduce incidence of re-tears | [9] |
| Intervertebral disc degeneration  (spinal fusion) | Murine | Adenoviral | Human BMP-2 | In vivo (mouse) | Bilaterally into paravertebral muscles of the lumbar spine. | Bone formation along with spinal fusion, biomechanical stabilization | [10] |
| Fracture  (tibial defect model) | Murine BM | AAV | Rat BMP2, Mouse VEGF | In vivo (mouse) | Tail vein | Supportive role during early bone formation, increased vascularity and osteoblastogenensis, homing, paracrine signals, enhanced bone formation | [11] |
| Cartilage repair (partial-thickness defects) | Ovine BM | Adenoviral | TGFβ | In vivo (sheep) | Transplanted into knee | Improved recovery | [12] |
| Osteoporosis  (osteopenia mouse) | Murine BM | AAV | Rat BMP-2, Mouse α4 Integrin | In vivo (mouse) | Tail vein | Influenced mobilization, homing, pro-osteoblastic mechanism, paracrine mechanisms like increased proliferation/cell recruitment, improved bone growth | [13] |
| Cartilage repair (osteochondral defects induced) | Murine skeletal muscle | Retroviral | Human BMP-2, VEGF, sFlt-1 | In vivo (rat) | Transplanted into knee | Functional improvement | [14] |
| Cartilage repair | Porcine BM | Adenoviral | Human TGFβ-1 | In vivo (mouse) | Implanted into subcutaneous tissue | Robust chondrogenic differentiation | [15] |
| Osteoarthritis  (MIA-induced) | Murine skeletal muscle | Retroviral | Human sFlt-1- and BMP-4 | In vivo (rat) | Intra-articular | Lower apoptosis, autocrine/paracrine effects, improved chondrogenic differentiation and recovery | [16] |
| Skull bone regeneration (cranial defects induced) | Swine BM | Adenoviral | Human BMP-2 | In vivo (swine) | N/A | Solid bone formation with well-mineralized bone | [17] |
| Tendinitis  (bilateral tendinitis lesions) | Horse BM | Adenoviral | IGF-1 | In vivo (horse) | Injected into lesion | Anti-inflammatory, functional recovery | [18] |

**Abbreviations:** AAV: Adeno-associated virus; BM: Bone marrow; BMP-2: Bone morphogenetic protein-2; BMP-4: Bone morphogenetic protein-4; CXCR3: C-X-C chemokine receptor type 3; IFN-γ: Interferon gamma; IGF-1: Insulin-like growth factor-1; IL-1: Interleukin 1; IL-10: Interleukin 10; IP-10: Interferon gamma-induced protein 10; MIA: Mono-iodoacetate; RGD: Arginylglycylaspartic acid; sFlt-1: Soluble fms-like tyrosine kinase-1; SOX-9: SRY (sex determining region Y)-box 9; TGFβ-1: Transforming growth factor beta-1; TNF-α: Tumor necrosis factor-alpha; UCB: Umbilical cord blood; VEGF: Vascular endothelial growth factor; ZNF145: zinc-finger protein145.

**RELATED REFERENCES**

1. Otsuru S, Gordon PL, Shimono K, Jethva R, Marino R, Phillips CL, Hofmann TJ, Veronesi E, Dominici M, Iwamoto M, Horwitz EM: **Transplanted bone marrow mononuclear cells and MSCs impart clinical benefit to children with osteogenesis imperfecta through different mechanisms**. *Blood* 2012, **120**:1933–1941.

2. Greish S: **Human umbilical cord mesenchymal stem cells as treatment of adjuvant rheumatoid arthritis in a rat model**. *World J Stem Cells* 2012, **4**:101.

3. Mao F, Xu W-R, Qian H, Zhu W, Yan Y-M, Shao Q-X, Xu H-X: **Immunosuppressive effects of mesenchymal stem cells in collagen-induced mouse arthritis**. *Inflamm Res* 2010, **59**:219–225.

4. Augello A, Tasso R, Negrini SM, Cancedda R, Pennesi G: **Cell therapy using allogeneic bone marrow mesenchymal stem cells prevents tissue damage in collagen-induced arthritis**. *Arthritis Rheum* 2007, **56**:1175–1186.

5. Moshaverinia A, Xu X, Chen C, Akiyama K, Snead ML, Shi S: **Dental mesenchymal stem cells encapsulated in an alginate hydrogel co-delivery microencapsulation system for cartilage regeneration**. *Acta Biomater* 2013, **9**:9343–9350.

6. Castro-Govea Y, Cervantes-Kardasch VH, Borrego-Soto G, Martínez-Rodríguez HG, Espinoza-Juarez M, Romero-Díaz V, Marino-Martínez IA, Robles-Zamora A, Álvarez-Lozano E, Padilla-Rivas GR, Ortiz-López R, Lara-Arias J, Vázquez-Juárez J, Rojas-Martínez A: **Human bone morphogenetic protein 2-transduced mesenchymal stem cells improve bone regeneration in a model of mandible distraction surgery**. *J Craniofac Surg* 2012, **23**:392–396.

7. Liu TM, Guo XM, Tan HS, Hui JH, Lim B, Lee EH: **Zinc-finger protein 145, acting as an upstream regulator of SOX9, improves the differentiation potential of human mesenchymal stem cells for cartilage regeneration and repair**. *Arthritis Rheum* 2011, **63**:2711–2720.

8. Cao L, Yang F, Liu G, Yu D, Li H, Fan Q, Gan Y, Tang T, Dai K: **The promotion of cartilage defect repair using adenovirus mediated Sox9 gene transfer of rabbit bone marrow mesenchymal stem cells**. *Biomaterials* 2011, **32**:3910–3920.

9. Gulotta LV, Kovacevic D, Packer JD, Deng XH, Rodeo SA: **Bone marrow-derived mesenchymal stem cells transduced with scleraxis improve rotator cuff healing in a rat model**. *Am J Sports Med* 2011, **39**:1282–1289.

10. Sheyn D, Rüthemann M, Mizrahi O, Kallai I, Zilberman Y, Tawackoli W, Kanim LEA, Zhao L, Bae H, Pelled G, Snedeker JG, Gazit D: **Genetically modified mesenchymal stem cells induce mechanically stable posterior spine fusion**. *Tissue Eng Part A* 2010, **16**:3679–3686.

11. Kumar S, Wan C, Ramaswamy G, Clemens TL, Ponnazhagan S: **Mesenchymal stem cells expressing osteogenic and angiogenic factors synergistically enhance bone formation in a mouse model of segmental bone defect**. *Mol Ther J Am Soc Gene Ther* 2010, **18**:1026–1034.

12. Ivkovic A, Pascher A, Hudetz D, Maticic D, Jelic M, Dickinson S, Loparic M, Haspl M, Windhager R, Pecina M: **Articular cartilage repair by genetically modified bone marrow aspirate in sheep**. *Gene Ther* 2010, **17**:779–789.

13. Kumar S, Nagy TR, Ponnazhagan S: **Therapeutic potential of genetically modified adult stem cells for osteopenia**. *Gene Ther* 2010, **17**:105–116.

14. Kubo S, Cooper GM, Matsumoto T, Phillippi JA, Corsi KA, Usas A, Li G, Fu FH, Huard J: **Blocking vascular endothelial growth factor with soluble Flt-1 improves the chondrogenic potential of mouse skeletal muscle-derived stem cells**. *Arthritis Rheum* 2009, **60**:155–165.

15. Xia W, Jin Y-Q, Kretlow JD, Liu W, Ding W, Sun H, Zhou G, Zhang W, Cao Y: **Adenoviral transduction of hTGF-beta1 enhances the chondrogenesis of bone marrow derived stromal cells**. *Biotechnol Lett* 2009, **31**:639–646.

16. Matsumoto T, Cooper GM, Gharaibeh B, Meszaros LB, Li G, Usas A, Fu FH, Huard J: **Cartilage repair in a rat model of osteoarthritis through intraarticular transplantation of muscle-derived stem cells expressing bone morphogenetic protein 4 and soluble Flt-1**. *Arthritis Rheum* 2009, **60**:1390–1405.

17. Chang SC-N, Lin T-M, Chung H-Y, Chen PK-T, Lin F-H, Lou J, Jeng L-B: **Large-scale bicortical skull bone regeneration using ex vivo replication-defective adenoviral-mediated bone morphogenetic protein-2 gene-transferred bone marrow stromal cells and composite biomaterials**. *Neurosurgery* 2009, **65**(6 Suppl):75–81; discussion 81–83.

18. Schnabel LV, Lynch ME, van der Meulen MCH, Yeager AE, Kornatowski MA, Nixon AJ: **Mesenchymal stem cells and insulin-like growth factor-I gene-enhanced mesenchymal stem cells improve structural aspects of healing in equine flexor digitorum superficialis tendons**. *J Orthop Res Off Publ Orthop Res Soc* 2009, **27**:1392–1398.
